# DFCP1 is a regulator of starvation-driven ATGL-mediated lipid droplet lipolysis

**DOI:** 10.1016/j.jlr.2024.100700

**Published:** 2024-11-19

**Authors:** Victoria A. Ismail, Meg Schuetz, Zak N. Baker, Jean A. Castillo-Badillo, Teri V. Naismith, David J. Pagliarini, David J. Kast

**Affiliations:** 1 Department of Cell Biology and Physiology, Washington University School of Medicine, St. Louis, Missouri, USA; 2 Howard Hughes Medical Institute, Chevy Chase, Maryland, USA; 3 Department of Biochemistry and Molecular Biophysics, Washington University School of Medicine, St. Louis, Missouri, USA; 4 Department of Genetics, Washington University School of Medicine, St. Louis, Missouri, USA

**Keywords:** *ZFYVE1*, DFCP1, ATGL, CGI-58, *ABHD5*, lipid, droplets, triglycerides, diglycerides, fatty acids, FRAP, *PNPLA2*

## Abstract

Lipid droplets (LDs) are transient lipid storage organelles that can be readily tapped to resupply cells with energy or lipid building blocks, and therefore play a central role in cellular metabolism. Double FYVE Domain Containing Protein 1 (DFCP1/*ZFYVE1*) has emerged as a key regulator of LD metabolism, where the nucleotide-dependent accumulation of DFCP1 on LDs influences their size, number, and dynamics. Here we show that DFCP1 regulates lipid metabolism by directly modulating the activity of Adipose Triglyceride Lipase (ATGL/*PNPLA2*), the rate-limiting lipase driving the catabolism of LDs. We show through pharmacological inhibition of key enzymes associated with LD metabolism that DFCP1 specifically regulates lipolysis and, to a lesser extent, lipophagy. Consistent with this observation, DFCP1 interacts with and recruits ATGL to LDs in starved cells, irrespective of other known regulatory factors of ATGL. We further establish that this interaction prevents dynamic disassociation of ATGL from LDs and thereby impedes the rate of LD lipolysis. Collectively, our findings indicate that DFCP1 is a nutrient-sensitive regulator of LD catabolism.

Lipid droplets (LDs) are transient lipid storage depots that provide lipids for the repair and biogenesis of membranous organelles, while also serving as a reservoir of energy for the cell. The breakdown of triacylglycerides (TAGs) from LDs into fatty acids (FAs) during nutrient stress plays a critical role in cell survival, and failure to do so contributes to the pathology of a host of metabolic diseases including lipodystrophies, obesity, insulin resistance, diabetes, NAFLD, and atherosclerosis (1). Thus, it is important for the cell to maintain tight control over LD metabolism via many regulatory proteins across key steps of LD growth and degradation.

The process of LD metabolism is dynamic and involves the packaging of TAGs into LDs (LD biogenesis) and the stimulated breakdown of the TAGs stored inside LDs to release FAs (LD catabolism) that are used by mitochondria. The latter stage is thought to be mediated by two sequential and intertwined mechanisms: the breakdown of large LDs by LD-associated lipases into small LDs (lipolysis), followed by efficient clearance by the autophagy-lysosomal pathway, in a distinct process known as lipophagy (2, 3). It is believed that the lipophagy pathway can only break down smaller LDs due to the limited size of the phagophore, and thus, cells that form larger LDs tend to favor lipolysis to mobilize FAs (4). It should be noted that a surplus of FAs can also be repackaged into LDs, which is important for protecting cells from the toxic accumulation of FAs in the cytosol.

During lipolysis, TAGs are sequentially hydrolyzed into diacylglycerol (DAG), monoacylglycerol (MAG), and glycerol, releasing an FA at each of these steps. The first step of this process—hydrolysis of TAG into DAG—is slow and requires the lipase adipose triglyceride lipase (ATGL). However, the process can be markedly enhanced through a combination of AMPK and/or PKA-dependent phosphorylation of ATGL (5, 6, 7), as well as through the interaction of ATGL with its coactivator 1-acylglycerol-3-phosphate O-acyltransferase ABHD5, which is more commonly known as Comparative gene identification-58, or CGI-58. (8). Aside from phosphorylation and regulation via a few key regulatory proteins, very little is known about the molecular factors that modulate ATGL’s lipolytic activity.

The autophagy-associated protein, DFCP1, has recently gained a lot of attention as a novel regulator of LD metabolism. Both endogenous (9) and overexpressed (9, 10, 11) DFCP1 has been shown to accumulate on LDs in cells, and the expression of DFCP1 correlates with increased LD size (10), whereas knockdown of DFCP1 was shown to do the opposite—it significantly decreased LD size while concomitantly increasing the number of LDs. This accumulation of LDs was also shown to be dependent on a novel bifunctional NTPase domain housed within DFCP1 that hydrolyzes both ATP and GTP (9). Additionally, it was proposed that DFCP1 is recruited along with Rab18 and Seipin to LDs through interaction with ZW10 (Centromere/kinetochore protein zw10 homolog) (12), a central component of the NRZ ER-LD contact site complex. However, it is unknown if DFCP1’s functions on LDs require the NRZ complex, or if DFCP1 can act as an autonomous tether between LDs and the ER.

The specific mechanism by which DFCP1 regulates LD size is unclear. It has been proposed that DFCP1 participates in the biogenesis of LDs shortly after the import of exogenous FAs. The addition of FAs has been shown to promote an autophagic response (13, 14), which leads to an increase in PI3P-rich autophagosome precursor sites in the ER. Consequently, DFCP1 accumulates at these sites due to its intrinsic ability to bind PI3P. While DFCP1 was shown to not influence autophagosome formation (9, 15), it may instead assist in the biogenesis of LDs at these same sites (16). Recently, DFCP1 has been shown to promote TAG storage in LDs by impairing LD turnover and FA metabolism (9). However, during times of nutrient stress, DFCP1 partially translocates away from LDs to sites of autophagosome formation in the ER in a manner that depends on its NTPase activity. This translocation is coincidentally met with an increase in LD turnover and FA metabolism (9). It is therefore tempting to speculate that under basal conditions, DFCP1 protects LDs from spurious lipolysis, and upon starvation, DFCP1 translocates away from LDs to promote their catabolism.

Here we show that DFCP1 is a regulator of the rate-limiting lipolysis enzyme, ATGL. Specifically, we show that ablation of DFCP1 profoundly influences LD turnover in cells that were pharmacologically inhibited in their ability to perform LD biogenesis and lipophagy. Moreover, we found that DFCP1 directly interacts with ATGL via the N-terminus of ATGL and the C-terminus of DFCP1, and the interaction is important for both the recruitment and the sequestration of ATGL to the LD surface. Conversely, ablation of DFCP1 markedly enhances ATGL dynamics on LDs and leads to a marked increase in the lipolysis of LDs. Overall, we demonstrate that DFCP1 primes lipolysis by anchoring and suppressing ATGL on the surface of LDs. Upon starvation and/or nucleotide hydrolysis, DFCP1 releases this repression and allows for ATGL to begin lipolysis.

## Materials and Methods

### Mammalian cell culture

U2OS, Hep3B, and 3T3L1 (preadipocyte) cells were cultured at 37°C with 5% CO_2_ and in growth media consisting of MEM GlutaMax or DMEM GlutaMax supplemented with 10% fetal bovine serum (FBS) and antibiotic-antimycotic (Thermo Fisher Scientific, Waltham, MA), respectively. Two days before live-cell imaging experiments, 50,000 cells were seeded on 3.5 cm imaging dishes and grown to 40% confluency before transfection with the indicated plasmids using either Lipofectamine 2000 (ThermoFisher Scientific) or FuGENE HD (Promega). In some cases, cells were also incubated for 2 days before imaging with a siRNA mixture, consisting of 5 μl of RNAiMax (ThermoFisher Scientific) and 2.5 pmol of the specified siRNA. Cells were induced to form LDs by Supplementing the growth media with 200 μM oleic acid (OA) dissolved in ethanol for 4 or 24 h (as indicated), before exchanging the media with either basal growth media (fed) or EBSS (starved) for 1, 4, or 18 h (as indicated) prior to imaging. LDs were identified by incubating with the LD-specific dye LipidTOX Deep Red (ThermoFisher Scientific) at a volume ratio of 1:10,000 for 30 min prior to imaging.

### Cloning

The generation of constructs used in this manuscript was performed as follows:

#### Mutations

In the DFCP1 (K193A and R266Q) and ATGL (D166G) were introduced using the QuikChange mutagenesis kit (Agilent, Santa Clara, CA).

#### BFP- and GFP-DFCP1 constructs

All DFCP1 constructs, including DFCP1 truncations, were cloned as previously described in (9).

#### FLAG-ATGL

Human ATGL (Uniprot ID: Q96AD5) was cloned into a custom vector, where the DNA sequence encoding GFP in a pEGFP-C1 vector was replaced with a DNA sequence encoding a FLAG-TEV sequence using the NheI and BglII restriction sites.

#### MBP-ATGL (1–254)

Residues 1–254 of human ATGL (Uniprot ID: Q96AD5) were inserted into the pMal-c2e bacterial expression vector using the EcoRI and Sal1 restriction enzyme cleavage sites.

#### EGFP- and mCherry-ATGL (1–504)

Full-length Human ATGL (Uniprot ID: Q96AD5) was subcloned into pEGFP-C1 and pmCherry-C1 mammalian expression vectors using the BglII and Sal1 restriction enzyme cleavage sites.

#### GFP-CGI-58

CGI-58 (Uniprot ID: Q9DBL9) was PCR amplified from cDNA from WT mouse brown fat pads, provided kindly by Dr Philipp Scherer, and cloned into a pEGFP-C1 vector using the SacI and SalI restriction sites.

### U2OS DFCP1 knockout cell line

The U2OS DFCP1 Knock-out cell line was generated using Alt-R® CRISPR/Cas9 system (IDT, Coraville, IA), as previously described (9). In brief, a custom guide RNA (UAGCAGUGAUCGAUACGGAA) was annealed to tracer RNA, bound to purified *S. pyrogenes* Cas9 nuclease (IDT), and electroporated into U2OS cells using an Amaxa 4D-Nucleofector (Lonza Biosciences, Basel, Switzerland). Electroporated cells were then sorted into 96 well plates and checked for DFCP1 expression after 72 h using western blotting. Clonal populations that were depleted of DFCP1 were further analyzed by next-generation sequencing and monoclonal populations were selected based on the presence of two different but equivalent levels of frame-shifting deletions (to ensure both alleles of DFCP1 were edited). Finally, western blotting was used to verify that the resulting clones were depleted of DFCP1.

### Live cell imaging and image analysis

All cells were imaged using either a Nikon Ti2 inverted microscope equipped with a 100× (1.4 NA) Plan-Apo oil immersion objective and a Yokogawa CSU-W1 spinning disk confocal attached to a Hamamatsu ORCA-FLASH4.0 CMOS camera. Cells in either DMEM FluoroBrite (ThermoFisher Scientific) medium supplemented with 5% FBS or EBSS lacking phenol red (MilliporeSigma) were imaged at 37°C and 5% CO_2_. Images stacks were captured at 16-bit 2048 × 2044 resolution with an axial spacing of 0.2 μm using the Nikon Elements Software package. All images used to quantify LD diameters and density were captured blindly and randomly, which involved imaging the nearest BFP-lifeAct or GFP expressing cell to a random set of x-y coordinates. All images were blinded prior to analysis by 1 experimenter and image analysis was performed using the software Fiji (https://imagej.net/Fiji) by another experiment. LD density and diameters were scored manually and 2D/3D colocalization analysis was performed using the Fiji Coloc2 analysis tool. Specifically, LD diameter was measured in the plane where a given LD’s diameter was the largest. Lipid droplet density was determined by dividing the total number of LDs by the cell volume, which was calculated by summing the area for each cell that includes the cytosolic GFP fluorescence in each imaging plane of a confocal Z-series and multiplying by the axial spacing (typically 0.2 μm).

### Cell fixation

U2OS cells expressing GFP-DFCP1^WT^ or GFP-DFCP1^KA^ were grown over no. 1.5 round glass coverslips (Electron Microscopy Sciences) and fixed in 4% w/v paraformaldehyde (Electron Microscopy Sciences) in PBS for 15 min at RT, rinsed with PBS, and permeabilized in 0.1% v/v Triton X-100 (Pierce Biotechnology). Following rinsing with PBS, 1:2000 LipidTOX was added along with 0.25 μg ml^−1^ of 4′,6-diamidino-2-phenylindole (DAPI; Molecular Probes) for 15 min, and added to cells for 30 min at RT. After rinsing with PBS, coverslips were mounted in a solution of ProLong Glass Antifade mountant (P36980, Invitrogen), and allowed to cure for 24 h before imaging.

### FRAP

Imaging was performed on an Andor Dragonfly spinning disk confocal system using an iXon Lite EMCCD camera and a Nikon Eclipse Ti2 inverted microscope. Localized photobleaching was performed with Andor Mosaic 3 DMD array using a 445 nm laser at 145 nW power was scanned across the designated region at 0.9 ms/μm^2^. For imaging, 405, 488, and 637 nm solid-state lasers were used along with BFP 480/20 nm, GFP 525/20 nm, and Far Red 700/20 nm emission filters. Images were obtained with a Leica 63×, 1.4 NA oil immersion objective. Photobleaching regions of interest was performed approximately 1 min into imaging. Image acquisition was performed every 5 s. All imaging was performed at 37°C and 5% CO_2_.

#### FRAP analysis

FRAP traces were normalized by setting the maximum pre-photobleach fluorescence intensity of GFP-ATGL within a given ROI to 1 and the minimum fluorescence intensity after photobleaching to 0. Individual traces of a given condition were aligned to the post-photobleached minimum and averaged. Average fluorescent recovery traces were fitted using a 2-component association model to account for the fast recovery of a small fraction of cytosolic ATGL surrounding the photobleached LD, as well as the recovery of GFP-ATGL to the LD.

$$F\left(t\right)=F_{R}\left[\;\chi \;\left(1-e^{-k_{f}t}\right)+\left(1-\chi \right)\left(1-e^{-ik_{s}t}\right)\right]$$

in this equation, *F*(*t*) is the normalized fluorescence intensity as a function of time, *F*_*R*_ is the maximum recovered fluorescent intensity prior to photobleaching (normalized to 1 in this analysis), χ is the mol fraction, *k*_*f*_ is the fast recovery rate, and *k*_*s*_ is the slow recovery rate. All fits were performed using a weighted least squares fitting routine on the first 13 min of the data, and the mol fraction was allowed to vary but restricted to a maximum of 1; both the fast and slow recovery rates were allowed to vary. Additionally, in the comparisons of the GFP-ATGL recoveries at 15 min, both the recovery plateau and the slow rate were unrestricted and allowed to vary. In the comparison of the slow rates, the mobile fraction was fixed to the projected recovery of GFP-ATGL in DFCP1 KO cells for all traces, and only the slow rate and mol fraction were allowed to vary.

### Double drug experiment

Hep3B cells seeded on D35 imaging dishess treated with either non-targeting (NT) or DFCP1-targeting siRNAs (KD). After 48 h, control and DFCP1 KD cells were transfected with GFP using FuGENE and then, 4 h later, treated with 200 μM OA for 4 h. The OA was removed by washing cells twice with PBS before placing cells in starvation media (EBSS) supplemented with either a combination of two LD metabolism drugs or an equivalent volume of DMSO. To study the impact of DFCP1 KD on LD biogenesis, both lipolysis and lipophagy were inhibited by treating starved cells with 20 μM ATGLi (Cayman #15284) and 50 μM Lalistat 2 (LALi; Cayman #25347). To study the impact of DFCP1 on lipolysis, both biogenesis and lipophagy were inhibited by treating starved cells with 10 μM DGAT1i (Sigma-Aldrich, PF-04620110) and 50 μM LALi. To study the effect of DFCP1 on lipophagy, both biogenesis and lipophagy were inhibited by treating starved cells with 10 μM DGAT1i and 20 μM ATGLi. Cells were treated with 1:10,000 LipidTOX Deep Red after 18 h incubation with the inhibitors and imaged.

### Cell-based TLC lipase assay

Cells were seeded on 10 cm dishes and then treated with a non-targeting siRNA or an siRNA for DFCP1 in complex with RNAimax. After 48 h, cells were washed with PBS and incubated with 3 μM BODIPY-C12 558/568 in EBSS for the durations indicated in relevant figure legends. Cells were harvested via scraping and spun down in PBS. Cellular lipids were extracted using chloroform and spotted on aluminum-backed silica plates (Sigma) and then developed using 1:2 cyclohexane:ethyl acetate. Plates were imaged using BioRad Imager and spots were quantified using ImageJ.

### Phosphorylation experiment

3T3L1 mouse preadipocytes were cultured to confluency in growth media (DMEM Glutamax with 10% FBS) and LD formation was stimulated by adding 200 μM OA to the media. After 20 h, media was removed, cells were washed with PBS, and cells were incubated in either fresh growth media (fed) or EBSS (starved) for 24 h. Prior to harvesting, cells were incubated with 0.1 μM calyculin A (LC Laboratories; C-3987) for 15 min at 37°C to inhibit serine phosphatases. Cells were then harvested in PBS by scraping and lysed in lysis buffer containing 1% Triton-X, 1X PMSF, 0.1 μM calyculin A, 1X protease inhibitors (Pierce protease inhibitor tablets, EDTA free; A32965), 1X phosphatase inhibitors (Pierce phosphatase inhibitor tablets; A32957), 20 mM Tris, pH 7.5) and freeze-thawed once with liquid nitrogen.

### Western blotting

Cells for western blotting were treated the same way as those for imaging, except, in this case, 200,000 cells were seeded onto a 6 cm plate one day before transfection with plasmids or siRNAs. Cells were harvested 3 days post-seeding in lysis buffer consisting of TBS supplemented with 1% Triton-X, 1 mM PMSF, and 1 X protease inhibitor cocktail (APExBIO). The lysis mixture was pipette mixed and incubated on ice for 30 min. The lysate was then clarified at 12,000 *g* for 10 min and the supernatant was mixed with 3X SDS sample buffer. Samples were run on 12% polyacrylamide gels and transferred onto Immobilon-P polyvinylidene difluoride (PVDF) membranes (MilliporeSigma) membrane in transfer buffer containing 10% ethanol and 0.01% SDS at 130 mA for 3 h at 4°C. Membranes were allowed to dry and then blocked for 1 h with 5% BSA in TBS Supplemented with 0.1% Tween (TBS-T), before incubating with the specified primary antibodies for 4 h to overnight at 4°C. Blots were then washed with TBS-T and incubated with horseradish peroxidase (HRP)-linked anti-mouse and anti-rabbit immunoglobulin G secondary antibodies for 30 min to 1 h at room temperature. Washed blots were developed using the clarity western ECL substrate (Bio-Rad Laboratories, Hercules, CA) and imaged using a Gel-Doc Imager (Bio-Rad). The relative abundance of proteins was determined by densitometry analysis using the program Fiji. The anti-DFCP1 (85156S), anti-ATGL (2138S), and anti-Calreticulin (12238S) antibodies were obtained from Cell Signaling Technologies (CST). The anti-phospho-ATGL (S406) (ab135093) antibody was obtained from Abcam. The anti-CGI-58 (00,041,386) antibody was obtained from ProteinTech. The anti-14-3-3 (sc-133233), anti-GFP (sc-9996), and anti-GAPDH (sc-365062) antibodies were obtained from Santa Cruz Biotechnologies. The anti-HIS (MA1 21,315) antibody was purchased from Invitrogen. The anti-Rab18 (A2812) antibody was purchased from ABClonal.

### Cell viability assays

U2OS cells were plated at an initial density of 300,000 cells/well in a 12-well plate, allowed to become 80%–100% confluent, and then treated with 200 μM of OA or palmitic acid (PA) for 20 h followed by 4 h or 24 h of growth media (DMEM with 10% FBS) or EBSS. Cells were washed with 1 ml of PBS and then lifted with 300 μl trypsin-EDTA followed by quenching the trypsin with 600 μl of growth media. Cells were pelleted at 300 *g* for 5 min and 600 μl of the supernatant was removed. The remaining 300 μl of solution was used to resuspend the cells. An equal volume of trypan blue was added, and living and dead cells were counted.

### Annexin V-FITC/PI staining

For annexin V staining, cells were incubated in 90 μg mL^−1^ annexin V in annexin V binding buffer consisting of 10 mM HEPES, pH 7.4, 140 mM sodium chloride, 2.5 mM CaCl_2_ for 15 min at room temperature prior to recording fluorescence on a Citation 5 microplate reader (Agilent-BioTek, Santa Clara, CA), exciting at 488 nm and measuring emission at 535 nm. For propidium iodide staining, cells were incubated in 500 μg mL^−1^ propidium iodide in annexin V binding buffer consisting of 10 mM HEPES, pH 7.4, 140 mM sodium chloride, 2.5 mM calcium chloride for 15 min at RT prior to examining fluorescence on a Citation 5 microplate reader (Agilent-BioTek, Santa Clara, CA), exciting at 535 nm and measuring emission at 610 nm. Measurements were normalized to DAPI fluorescence after fixation with methanol.

### Protein purification

#### FLAG-ATGL purification

Human FLAG-ATGL (Uniprot ID: Q96AD5) was expressed in 20 15-cm dishes of HEK 293T cells were cultured in DMEM (ThermoFisher Scientific) with 5% FBS and then transiently transfected with a solution of 1 μg mL^-1^ plasmid DNA and 1 mg mL^−1^ PEI. After 48 h expression, cells were harvested in PBS, pelleted, and resuspended in lysis buffer comprised of 25 mM Tris-HCl (pH 7.0), 150 mM NaCl, 1% Triton-X, 1 mM PMSF, and a protease inhibitor cocktail (ThermoFisher Scientific). Cells were incubated in lysis buffer on ice for 30 min and insoluble cellular components were removed by centrifugation at 13,000 *g* for 10 min. Clarified lysates were incubated with 1 ml Anti-DYKDDDDK (FLAG) Affinity Resin (Genscript) for 2 h at 4°C and then washed with 10 column volumes (CVs) of wash buffer, comprised of 25 mM Tris, pH 7.0, 150 mM NaCl, and 1 mM PMSF, followed by 10 CVs of wash buffer supplemented with 1 mM ATP. Bound ATGL was eluted by 4 sequential 1 h incubations of the FLAG resin with 1 CV of wash buffer supplemented with 1 mg mL^-1^ FLAG peptide (prepared in house). The elution fractions were pooled, and the excess FLAG peptide was removed by dialysis using a 14 kDa MWCO dialysis tubing in a buffer composed of 20 mM Tris-HCl (pH 7.5), 150 mM NaCl and 1 mM DTT. The dialyzed protein was spin concentrated using a 30 kDa MWCO ultracentrifugation filter (MilliporeSigma) and subjected to size exclusion chromatography using a Superose 6 attached to an AKTA Pure 25L (Cytiva Life Sciences) equilibrated in a buffer consisting of 20 mM Tris pH 7, 150 mM NaCl, and 1 mM DTT. The resulting peaks including ATGL were isolated together, spin concentrated using a 30 kDa MWCO ultracentrifugation filter, and flash frozen in liquid nitrogen.

#### MBP-ATGL (1–254) purification

The MBP-ATGL (1–254) (Uniprot ID: Q96AD5) construct was transformed into *Rosetta* (DE3) cells (Novagen) that were subsequently cultured in Terrific Broth medium that was supplemented with 100 μg mL^−1^ carbenicillin and 100 μg mL^−1^ chloramphenicol and incubated on a shaker at 37°C. Protein expression was induced with 1 mM IPTG at 18°C for 16 h. Cells were homogenized in lysis buffer, comprised of 25 mM Tris pH 8.0, 300 mM NaCl, 4 mM benzamidine hydrochloride, and 1 mM PMSF, and lysed using a microfluidizer 110L (Microfluidics, Newton, MA). Lysates were clarified by centrifugation at 16,000 *g* for 45 min and the supernatant was loaded onto amylose affinity resin (New England BioLabs). The column was washed with 10 CVs of lysis buffer and the protein was eluted with sequentially added 1 CV aliquots of lysis buffer supplemented with 20 mM maltose. Aliquots containing the eluted protein were combined and passed over a HiLoad 26/600 Superdex 200 pg size exclusion column (Cytiva) equilibrated in 20 mM Tris pH 8.0, 150 mM NaCl, and 1 mM DTT. The peak containing MBP-ATGL (1–254) was isolated, and the protein was spin-concentrated to a 2 ml volume using a 10 MWCO ultracentrifugation filter. The protein was further purified with a MonoQ 4.6/100 PE column (Cytiva), using a 100 mM–500 mM NaCl gradient spanning 30 CVs. The peak corresponding to MBP-ATGL (1–254) was isolated, spin concentrated, and then flash frozen in liquid nitrogen.

#### GFP nanobody purification

The His-GFP-nanobody construct (17) was transformed into *BL21* (DE3) cells (Novagen) that were subsequently cultured in Terrific Broth medium that was supplemented with 100 μg mL^−1^ carbenicillin and incubated on a shaker at 37°C. Protein expression was induced with 1 mM IPTG at 18°C for 16 h. Cells were homogenized in lysis buffer, comprised of 20 mM Tris pH 7.0, 300 mM NaCl, 4 mM benzamidine hydrochloride, and 1 mM PMSF, and lysed using a microfluidizer 110L (Microfluidics) at 4°C. Lysates were clarified by centrifugation at 12,000 *g* for 45 min and the supernatant was loaded onto 1 ml of Nickel-NTA resin after adding imidazole to a final concentration of 10 mM. The Nickel-NTA resin was washed with 10 CV of lysis buffer supplemented with 25 mM imidazole buffer. GFP-nanobody was eluted with lysis buffer supplemented with 250 mM imidazole buffer and then dialyzed overnight into PBS and conjugated to 1 g of (N-hydroxysuccinimide) NHS-activated agarose beads (Pierce) overnight at 4°C on a rocker. The reaction was quenched with 1M Tris, pH 8.0 for 15 min at RT. Conjugation efficiency was determined to be ∼95% and the concentration of GFP nanobody on the beads was determined to be 6 mg mL^−1^.

### Lipid droplet isolation

Control or DFCP1 KO cells were grown to confluence in 15 cm dishes and treated with 200 μM OA overnight to promote the production of LDs. The cells were then harvested and mechanically lysed using a 28-gauge needle in a hypotonic solution containing 20 mM Tris-HCl (pH 7.5), 1 mM NaF, 1 mM PMSF, protease inhibitor cocktail). Cell lysates were clarified by low-speed centrifugation at 1,000 *g* (4°C) for 10 min and the resulting supernatants were mixed with an equal volume of ice-cold lysis buffer containing 20% (v/v) sucrose. These diluted cell lysates were then added to an ultracentrifuge tube, layered with 5 ml ice-cold lysis buffer containing 5% sucrose and further layered with enough ice-cold lysis buffer to fill the tube. The final solution was centrifuged for at 28,000 *g* (4°C) for 1 h and the floating LD layer was collected and placed in a microfuge tube. The floating layer was again centrifuged at 20,000 *g* (4°C) for 10 min, the bottom soluble fraction was removed, and the process was repeated until the LD layer was reduced to a final volume of 50 μl.

### In vitro TLC lipase assay

HEK 293T cells on 15 cm dishes were transfected with 3.33 μg of each construct for a final 10 μg of total DNA using 25 μl polyethylenimine (PEI; Polysciences, Warrington, PA). After 2 days, cells were lysed mechanically using a 28-gauge needle in a hypotonic solution containing 20 mM Tris-HCl (pH 7.5) 1 mM PMSF and protease inhibitor cocktail). HEK 293T lysates were clarified at 13,000 *g* for 10 min. Clarified lysates were normalized by total protein, determined by a Bradford assay, and then 500 μg of lysate was incubated with 10 μl of LDs, isolated from either control or DFCP1-KO cells, for the indicated length of time at 37°C. After incubation, lipids were extracted with chloroform. The organic, solid, and aqueous phases were separated by spinning at 21,000 *g* for 10 min at 4°C, and the organic phase was isolated. Separation of lipid species was achieved by spotting 1 μl of the solution on aluminum-backed silica plates (Sigma) and the plates were then developed using 70:30:1 hexane:diethyl ether:acetic acid. Visualization was done by brief incubation in 10% cupric sulfate in 8% aqueous phosphoric acid, followed by drying for 10 min at RT and then heating on a hot plate at 160°C. Plates were imaged using a LI-COR imager and spots were quantified using ImageJ.

### Lipidomic profiles of LDs purified from control and KO cells

Bligh-Dyer extraction was performed to extract TAG, PC, and CE from LD samples. The TAG (17:1/17:1/17:1), DAG (21:0/21:0/21:0), and d7-PE (18:2) were used as internal standards for TAG, DAG, and CE, respectively. The internal standard was added to the samples before extraction. Analysis of TAG, DAG, and CE was performed with a Shimadzu 20A HPLC system coupled to an API4000 mass spectrometer operated in positive multiple reaction monitoring (MRM) mode. Quality control (QC) samples were prepared by pooling aliquots of the study samples and were used to monitor the instrument performance. The QC samples were injected between every 4 study samples. Only the lipid species with a coefficient of variance of less than 15% in QC injections are reported. The relative quantification of lipids was provided, and the data were reported as the peak area ratios of the analytes to the corresponding internal standards. The relative quantification data generated in the same batch are appropriate to compare the change of an analyte in a test sample relative to other samples (e.g., control vs. treated, or samples in a time-course study).

### Lipidomic lipolysis rate assay

HEK 293T cells on 15 cm dishes were transfected with 3.33 μg of each construct for a final 10 μg of total DNA using 30 μl polyethylenimine (PEI; Polysciences). Cells were lysed mechanically in a hypotonic solution (20 mM Tris-HCl (pH 7.4), 1 mM EDTA, 1 mM NaF, 1 mM PMSF and protease inhibitor cocktail) 2 days post-transfection using a 28-gauge needle. HEK 293T lysates were clarified at 13,000 *g* for 10 min. Clarified lysates were normalized by total protein, determined by a Bradford assay, and then 650 μg of lysate was incubated with 20 μl of LDs isolated from DFCP1 KO cells for the indicated length of time at 4°C. After incubation, lipids were extracted using a Bligh-Dyer method with the addition d4-palmitoyl-ethanolamide (Cayman Chemical) as an internal standard at a final concentration of 100 nM. Extracted lipids were separated on an Acquity CSH C18 column (100 mm × 2.1 mm x 1.7 μm particle size; Waters) at 50°C using the following gradient: 2% mobile phase B from 0-2 min, increased to 30% B over next 1 min, increased to 50% B over next 1 min, increased to 85% over next 14 min, increased to 99% B over next 1 min, then held at 99% B for next 7 min (400 μl/min flow rate). Column re-equilibration of 2% B for 1.75 min occurred between samples. For each analysis 1 μl/sample was injected by the autosampler. QC samples were injected at regular intervals to ensure constancy across runs. Mobile phase A consisted of 10 mM ammonium acetate (Sigma Aldrich) in 70% (v/v) ACN 30% (v/v) water with 250 μl/L acetic acid (Sigma Aldrich). Mobile phase B consisted of 10 mM ammonium acetate in 90% (v/v) IPA 10% (v/v) ACN with 250 μl/L acetic acid.

MS acquisition was performed by a Thermo Exploris 240 Orbitrap mass spectrometer. Samples were ionized by a (HESI II) source (Thermo Scientific) kept at a vaporizer temperature of 350°C. Sheath gas was set to 50 units, auxiliary gas to 8 units, sweep gas to 1 unit. MS was operated in polarity switching mode with the spray voltage set to 3,500 V for positive mode and 2,500 V for negative mode. The inlet ion transfer tube temperature was kept at 325°C with 70% RF lens. Full MS1 scans were acquired at 22,500 resolution (at 200 m/z), max ion accumulation time of 100 ms, with a scan range of m/z 200 to 1,600. MS2 scans (Top 3) were acquired at 30,000 resolution (at 200 m/z), max ion accumulation time of 50 ms, 1.0 m/z isolation window, stepped normalized collision energy (NCE) at 20, 30, 40, and a 10.0 s dynamic exclusion. Automatic gain control (AGC) targets were set to standard mode for both MS1 and MS2 acquisitions. LC-MS files for lipidomics were processed using Compound Discoverer 3.3 (Thermo Scientific) and LipiDex (18). All peaks with a 1.4–23 min retention time and 100 Da–5,000 Da MS1 precursor mass were aggregated into compound groups using a 10 ppm mass tolerance and 0.4 min retention time tolerance. Peaks were excluded if peak intensity was less than 2 × 10^6^, peak width was greater than 0.75 min, signal-to-noise ratio was less than 1.5, or intensity was < 3-fold greater than blank. MS2 spectra were searched against an in silico-generated spectral library. Spectra matches with a dot product score >500 and reverse dot product score > 700 were retained for further analysis. Lipid MS/MS spectra that contained <75% interference from co-eluting isobaric lipids, eluted within a 3.5 median absolute retention time deviation (M.A.D. RT) of each other and were found within at least 2 processed files were used. If individual fatty acid substituents were unresolved, then identifications were made with the sum of the fatty acid substituents. Lipid identifications were filtered with the Lipid Degreaser module within LipidDex2 (v0.1.0), based on retention time modeling. The retention time tolerance used was 0.5 min. Unreliable identifications were discarded. All lipids within a given trial of a given experiment were normalized to by the total spectra from PC and PE, which are two abundant lipids found in LDs from OA-stimulated cells and are not processed by ATGL and CGI-58.

### Statistical methods

Statistical comparisons of all measurements were performed using modifications (parametric, non-parametric, paired, or unpaired) of the student *t* test as indicated in the figure legend for each figure. Non-parametric statistical tests were employed when the distribution of at least one of the compared data sets did not obey normality. All statistical analyses were performed with the application Prism (GraphPad Software).

## Results

### DFCP1 inhibits LD catabolism

**Fig. 1:**
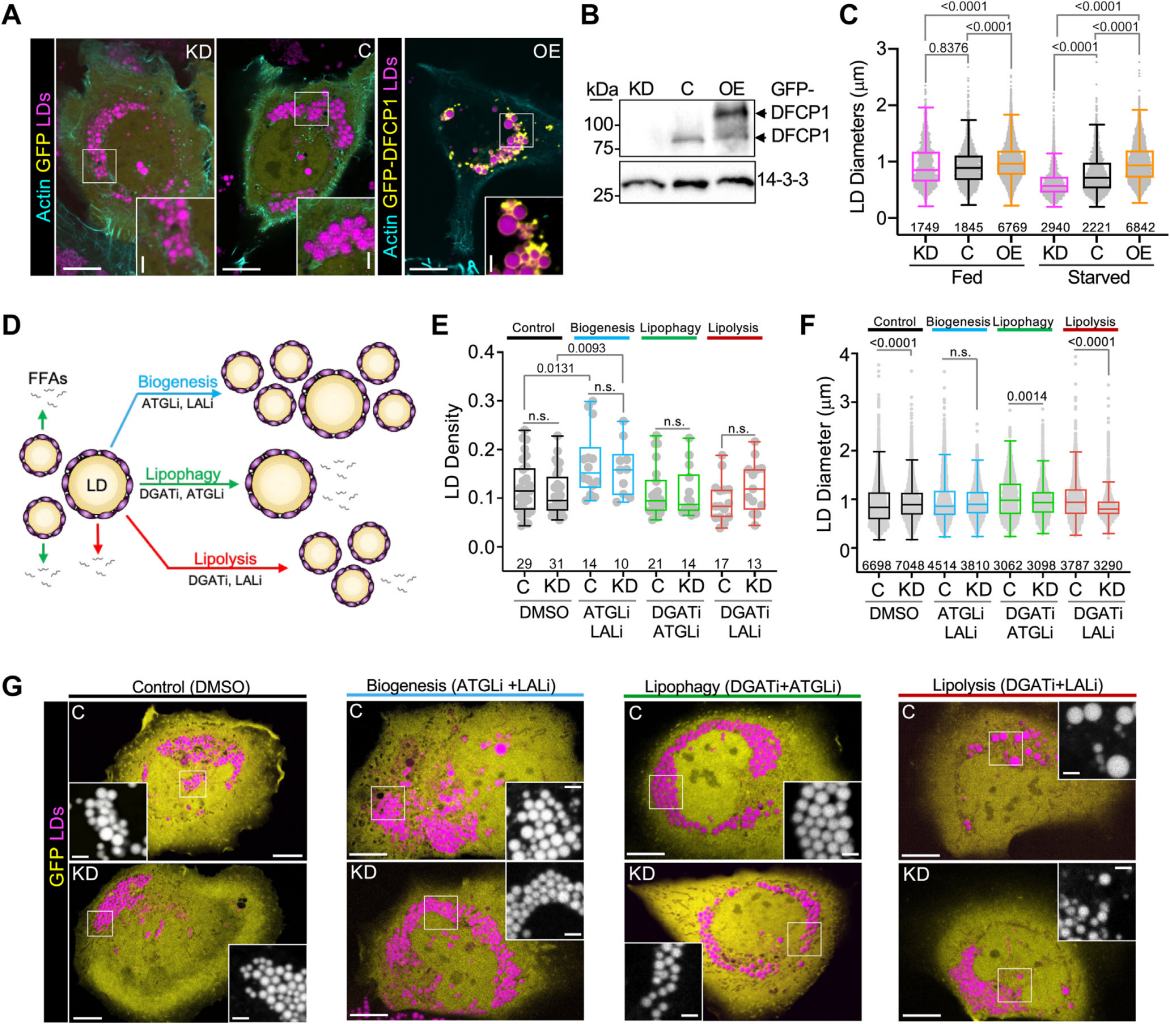
DFCP1 Inhibits LD Catabolism. A: Live cell confocal images of U2OS cells treated with DFCP1 siRNA (KD) or non-targeting siRNA treatment (control or “C”), expressing GFP-DFCP1 or GFP (yellow), and BFP-LifeAct (cyan). Cells were treated with 200 μM oleic acid (OA) for 20 h to stimulate LD formation. Prior to imaging, cells were incubated for 4 h in starvation media (EBSS) and then stained with LipidTOX Deep Red for 30 min. B: Western blot showing the extent of DFCP1 KD and overexpression of GFP-DFCP1 in the representative U2OS cells shown in (A). C: LD diameter distributions measured from the from the cells treated in (A). D: Diagram of LD growth and degradation pathways, including the combinations of small molecule inhibitors used to isolate specific pathways. E: Number of LDs/cell volume (density) quantified from images of control and DFCP1 KD cells expressing GFP that were treated with 200 μM OA for 20 h and starved (EBSS) for 18 h along with DMSO (vehicle) or the indicated inhibitors. F: LD diameter quantified from images of cells that were treated as in (E). G: Representative images of cells quantified in (E and F). The scale bars in whole-cell and inset images (A and G) represent 10 and 2 μm, respectively. The statistical significance of the measurements in (C and F) were determined using an unpaired non-parametric student *t* test (Mann–Whitney *U* test) on the indicated number of observations from two independent transfections. The statistical significance of the measurements in (E) were determined using an unpaired parametric student *t* test on the indicated number of observations from two independent transfections. Exact *P*-values are reported with exception to *P* > 0.05, which is not considered significant (n.s.).

DFCP1 has been implicated in regulating LD biogenesis (10, 16) and catabolism (9) where the knockdown (KD) of DFCP1 with siRNA is associated with smaller, more numerous LDs. Interestingly, we noted that in U2OS cells, stimulated with 200 μM oleic acid (OA), LD diameters increase as DFCP1 levels increase in the cell (Fig. 1A–C), particularly when cells are starved of amino acids by replacing the growth medium with EBSS for 4 or more hours (hereafter referred to as “starved”) – a condition known to drive LD catabolism. This effect is exacerbated when DFCP1 is overexpressed and suggests that DFCP1 inhibits the degradation of large LDs during starvation. However, given that LD biogenesis and the two canonical catabolic pathways, lipolysis, and lipophagy occur simultaneously, it is also possible that DFCP1 promotes biogenesis and/or accelerates the maturation of LDs in cells as previously suggested (16). Therefore, it is difficult to determine the precise role of DFCP1 in LD metabolism without systematically examining the impact of DFCP1 on biogenesis, lipolysis, and lipophagy.

To address this question, we examined the effect of knocking down DFCP1 on the number and size of LDs in response to treating cells with a combination of well-established small molecule inhibitors for diglyceride acyltransferase 1 (DGAT1), adipose triglyceride lipase (ATGL) and/or lysosomal acid lipase (LAL), which are key enzymes involved in LD biogenesis, lipolysis or lipophagy, respectively (19, 20, 21). Inhibiting DGAT1 with the DGAT1 inhibitor (DGATi) impairs the formation of LDs under all conditions. However, when cells are starved, DGAT1 inhibition mainly prevents the accumulation of small LDs that result from macroautophagy-mediated LD biogenesis (22), thereby leading to an average diameter that is larger than those found in vehicle-treated cells (supplemental Fig. S1A, B). Inhibition of ATGL with the ATGL inhibitor (ATGLi) impairs the first step in the hydrolysis of TAGs, and as a consequence, results in large LDs without impacting the number of LDs (4). In starved cells, we found that inhibition of ATGL produced the characteristic increase in LD diameter, but we also observed a significant reduction LD density (supplemental Fig. S1A, B), presumably because smaller LDs can still be removed through lipophagy. Inhibition of LAL with the LAL inhibitor (LALi) will impair autophagy- and/or lysosomal-dependent removal of LDs, and as a consequence lead to the accumulation of LDs under basal conditions without impacting their size (4). Under starved conditions, we observed a modest increase in LD density without an increase in LD size (supplemental Fig. S1A, B). It should be noted that the concentrations of LALi used in this study are the same as those used in other LD studies in liver cells (4), but this concentration has also been suggested to impair other cytosolic lipases in primary cells (23). However, given the mild impact on the LD size distribution in our studies, it is likely that we are not using a concentration that significantly impacts these off-target lipases.

The application of a single inhibitor, however, is not particularly informative because blocking one LD metabolic pathway may have a phenotypically similar outcome as blocking another. However, by multiplexing these inhibitors, it is possible to examine the impact of DFCP1 specifically on biogenesis, lipophagy, or lipolysis (Fig. 1D). Indeed, in control cells (cells treated with a non-targeting siRNA) that were starved for 4 h after a 24 h treatment of OA in conjunction with ATGLi and LALi, had a significantly higher density of LDs (number of LDs normalized by the volume of the cell) but the diameters of these LDs were unchanged from vehicle (DMSO) treated cells (Fig. 1E–G). This indicates that under these conditions, cells can generate LDs, but the mechanisms to reduce their size or remove them have been stalled. By contrast, starved control cells treated with OA in combination with DGATi and either ATGLi or LALi (Fig. 1E–G) led to a small decrease in LD density, but a marked increase in diameters of these LDs when compared to DMSO-treated cells, which is consistent with blocking biogenesis (reduced number) and one of the catabolic processes (increased size).

Having established that we could selectively monitor one pathway of LD metabolism at a time, we then examined the impact of these combinations of inhibitors on starved OA-treated cells where DFCP1 was knocked down. In starved KD cells treated with a combination of OA, ATGLi, and LALi, there was not a significant change in LD density or diameter when compared to control cells treated in the same way (Fig. 1E–G). This was quite surprising since DFCP1 has been previously implicated in LD biogenesis (16). Thus, we focused our attention on the catabolic pathways. When we investigated lipophagy by blocking biogenesis in conjunction with lipolysis, using the DGATi and ATGLi inhibitors, we did not observe a difference in LD density (Fig. 1E), when compared to control cells that were treated with the same conditions, but we did observe a small but significant decrease in the distribution of LD diameters (Fig. 1E, F). This is consistent with our previous observations on lipophagy, where we observed that DFCP1 KD reduced the number of LDs associated with autophagosomes, which results in the accumulation of smaller LDs (9). Finally, when we examined the impact of DFCP1 KD on lipolysis by blocking both biogenesis and lipophagy with DGATi and LALi, respectively, we found that DFCP1 KD cells (Fig. 1G) exhibited a higher density of LDs, that were significantly smaller than those in control treated cells (Fig. 1E, F). This latter observation suggests that DFCP1 may function early in the pathway of LD catabolism, where it could protect LDs from lipolysis, which would in turn impair lipophagy. For this reason, we decided to focus our attention on defining the role of DFCP1 in lipolysis.

### DFCP1 controls ATGL Localization in Cells

**Fig. 2:**
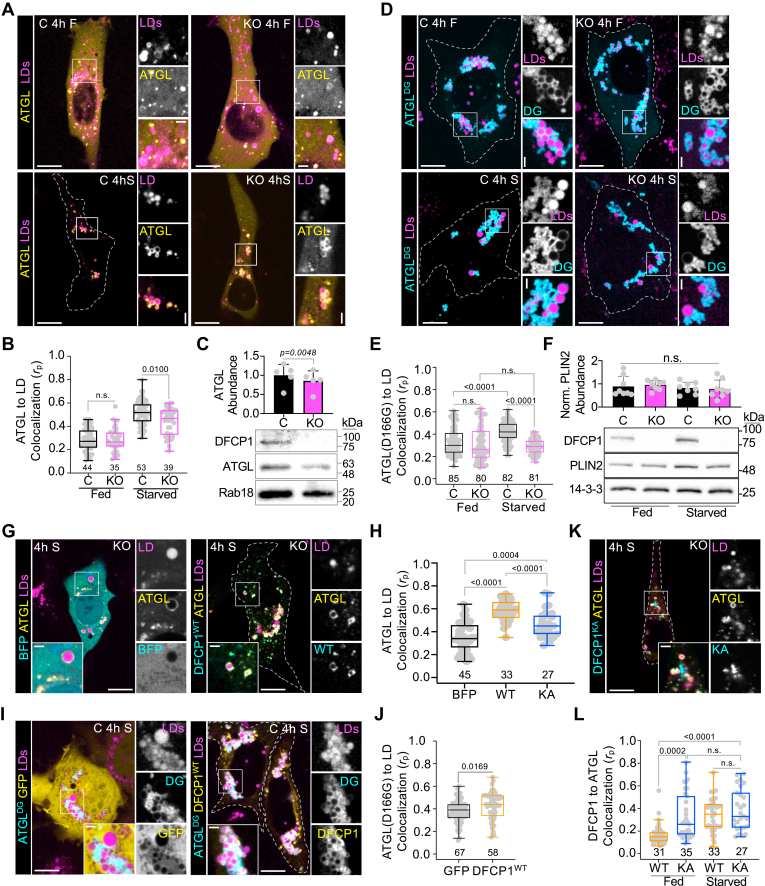
DFCP1 Controls ATGL Localization in Cells. A: Representative images of U2OS control (C) and DFCP1 CRISPR KO cells (KO) expressing GFP-ATGL and treated with 200 μM OA for 20 h. Cells were then fed (top row) or starved (bottom row) for 4 h before treating with LipidTOX Deep Red for 30 min prior to imaging. B: Colocalization analysis (Pearson’s correlation coefficient) of GFP-ATGL with LDs in control (black) and KO (magenta) cells described in A. C: Western blot of LDs purified from control (C, black) and DFCP1 KO (KO, magenta) U2OS cells showing the abundance of ATGL relative to the established LD associated protein, Rab18, and normalized to the mean abundance of ATGL found in the control samples. D: Representative images of U2OS control and DFCP1 KO cells expressing the lipase-dead mutant (D166G) of ATGL (BFP-ATGL^DG^). Cells were treated with OA and starved as in (A). E: Colocalization analysis of GFP-ATGL^DG^ with LDs in control (black) and KO (magenta) cells described in (D). F: Representative western blots showing PLIN2 expression relative to 14-3-3 expression in fed and starved control (C, black) and DFCP1 KO (KO, magenta) clarified U2OS cell lysates. (G) Representative images of U2OS cells expressing GFP-ATGL and either BFP (left) or BFP-DFCP1^WT^ (right). Cells were treated with OA and starved as in (A). H: Colocalization analysis of GFP-ATGL with LDs in cells described in (G and K) that were transfected with GFP-ATGL and BFP (black), BFP-DFCP1^WT^ (WT, orange) or BFP-DFCP1^KA^ (KA, blue). I: Representative images of U2OS cells expressing the lipase-dead mutant (D166G) of ATGL (BFP-ATGL^DG^) with either GFP (left) or GFP-DFCP1^WT^ (right). Cells were treated with OA and starved as in (A). J: Colocalization analysis of BFP-ATGL^DG^ with LDs in cells described in I. K: Representative image of U2OS cells expressing BFP-DFCP1^KA^. Cells were treated with OA and starved as in (A). L: Colocalization of GFP-ATGL with either BFP-DFCP1^WT^ (WT, orange) or BFP-DFCP1^KA^ (KA, blue) in the cells described in (K). The scale bars in whole-cell and inset images represent 10 and 2 μm, respectively. The statistical significance of the measurements in (B, E, H, J, and L) was determined using an unpaired parametric student *t* test on the indicated number of observations from two independent transfections. The statistical significance of the measurements in (C and F), was determined using an unpaired parametric student *t* test on 5 independent LD isolations for (C) and 3 independent experiments for each condition in (F) (all technical replicates are shown). Exact *P*-values are reported with exception to *P* > 0.05, which is not considered to be significant (n.s.).

LD lipolysis involves the hydrolysis of TAGs into FFAs and glycerol derivatives through the sequential activities of ATGL, hormone-sensitive lipase (HSL), and monoglyceride lipase (MGL). Among these enzymes, ATGL is known to be rate-limiting, and thus factors that regulate ATGL often have a profound impact on lipid storage. Interestingly, DFCP1 was found to be in in the proximity-proteome of ATGL, but not PLIN2 (24), which may indicate that DFCP1 is not simply an LD-associated protein but may function with ATGL in some capacity. To gain insight into this possibility, we examined the localization of ATGL to LDs as a function of nutrient conditions and DFCP1 expression. For this purpose, we silenced DFCP1 in U2OS cells using CRISPR/Cas9. This deletion completely abolished all DFCP1 protein expression (supplemental Fig. S1C) but did not significantly impact the viability (supplemental Fig. S1D) or the number of cells entering early (annexin V) or late (propidium iodide) apoptosis (supplemental Fig. S1E) when exposed to exogenous oleic (OA) or palmitic acid (PA) under fed or starved conditions. Using these cells, we found that depletion of DFCP1 had little effect on the localization of GFP-ATGL to LDs in fed cells that were treated with 200 μM OA for 20 h, but profoundly diminished the colocalization of ATGL with LDs in starved cells (Fig. 2A, B). Consistent with this observation, LDs isolated from DFCP1 KO cells also had a small (∼20%) but significant reduction in the amount of bound endogenous ATGL when compared to LDs isolated from control cells (Fig. 2C and supplemental Fig. S2A). The localization of ATGL is known to depend on its catalytic activity (6, 25), and we therefore wanted to determine if DFCP1 could impact the localization of an enzymatically dead variant of ATGL caring the D166G point mutation (ATGL^DG^). However, similar to GFP-ATGL, the colocalization of BFP-ATGL^DG^ with LDs was not influenced by DFCP1 in fed cells (Fig. 2D, E) and showed significantly less colocalization with LDs in starved OA-stimulated DFCP1 KO cells compared to control cells. This suggests that DFCP1-dependent localization of ATGL does not depend on its catalytic activity of ATGL.

In cells, the localization and function of ATGL is known to involve both phosphorylation of critical residues in ATGL’s lipase domain as well as interactions with PLINs. We therefore wanted to determine if DFCP1 was influencing ATGL’s localization by modulating 1 or more of these factors. Phosphorylation of S406 on ATGL by either AMPK or PKA was shown to be important for the dynamic mobilization of ATGL on to LDs (6, 25). Both kinases are activated in response to energetic stress, and therefore could explain why DFCP1 is particularly influential in blocking ATGL’s access to LDs during starvation. As commercially available primary antibodies sensitive to S406 phosphorylation are only available for mouse ATGL, we probed for S406 phosphorylation in control and DFCP1 CRISPR KO C2C12 and 3T3L1 cells (supplemental Fig. S2B, C). In both cell lines we observed a starvation-dependent increase in phosphorylation of endogenous mouse ATGL, however there was no observable difference to the extent of this phosphorylation between control and KO mouse cells. In addition to AMPK- and/or PKA-dependent phosphorylation, the PLIN protein networks have been shown to be important for regulating ATGL’s access to the LDs (26, 27, 28). It is generally believed that PLIN turnover by chaperon mediated autophagy helps promote ATGL-dependent lipolysis (29). Therefore, we questioned if DFCP1, which has been historically connected to the autophagy pathway, plays a role in regulating the abundance of PLINs on LDs during starvation. However, like ATGL phosphorylation, we did not see a significant difference in the accumulation of the three major PLINs expressed in U2OS cells (PLIN2, PLIN3 and to lesser extent PLIN5) in either fed or starved control or DFCP1 KO cells (Fig. 2F and supplemental Fig. S2D). Thus, DFCP1 appears to control ATGL localization irrespective of other regulatory factors.

Since DFCP1 ablation directly impaired the localization of ATGL to LDs, it is possible that overexpression of DFCP1 could enhance the localization of ATGL to LDs. To test this hypothesis, we transiently overexpressed BFP- or GFP-DFCP1 along with either GFP-ATGL (Fig. 2G and supplemental Fig. S2E) or BFP-ATGL^DG^ (Fig. 2I), respectively, in OA-treated U2OS cells that were either fed or starved. In these cells, we found the localization of both ATGL and ATGL^DG^ to LDs increased with DFCP1 expression when compared to cells overexpressing BFP or GFP, respectively (Fig. 2H, J). This DFCP1-dependent recruitment of ATGL to LDs was also dependent on the nutritional status of the cell since we saw more colocalization of GFP-ATGL with LDs in BFP-DFCP1^WT^ cells that were starved (Fig. 2G and H) when compared to those that were fed (supplemental Fig. S2E, F). Coincidently, we also observed an increase in the localization of BFP-DFCP1^WT^ to LDs in starved cells, when compared to fed cells expressing GFP-ATGL (supplemental Fig. S2G). This is particularly surprising, because we have previously shown that starvation partially redistributes DFCP1 from LDs to sites of autophagosome biogenesis (9) in the absence of ectopic expression of ATGL, and thus suggests that increased expression of either protein may mutually reinforce their localization to LDs during starvation.

This DFCP1-dependent localization of ATGL to LDs could arise directly through the localization of DFCP1, or through a DFCP1-dependent change to the pool of LDs, which may make them more “available” to ATGL. To distinguish between these possibilities, we exploited the observation that the localization of DFCP1 to LDs depends on its interaction with nucleotides (9). In particular, we have previously shown that interfering with the ability of DFCP1 to bind nucleotide through introduction of a point mutation (K193A) in the nucleotide binding domain of DFCP1 (DFCP1^KA^) also impaired the ability of DFCP1 to translocate from the ER on to LDs in Hep3B cells (9). This is also the case for U2OS cells (supplemental Fig. S2H, I). Thus, if ATGL localization depends on DFCP1 localization, we would expect to see less recruitment of ATGL to LDs in cells expressing the K193A mutant. Indeed, overexpression of BFP-DFCP1^KA^ resulted in significantly lower colocalization of ATGL to LDs when compared to those cells expressing BFP-DFCP1^WT^ (Fig. 2K, H). Importantly, GFP-ATGL colocalized equally well to both BFP-DFCP1^WT^ and BFP-DFCP1^KA^ in starved cells (Fig. 2L) despite showing less LD localization (Fig. 2H), which suggests that ATGL is either recruited to sites of DFCP1 accumulation, regardless of its location (LDs or ER), or is sensitive to a nucleotide-dependent structural state of DFCP1. Interestingly, this ATGL recruitment effect was also sensitive to starvation since there was not a statistically significant difference in the recruitment of ATGL to LDs in the presence of either BFP-DFCP1^WT^ or BFP-DFCP1^KA^ under fed conditions (supplemental Fig. S2E, F). Finally, unlike the case for DFCP1^WT^, this mutant did not show an increase in LD localization with GFP-ATGL expression (supplemental Fig. S2G), which is consistent with our previous observations (9).

### DFCP1 interacts with ATGL

**Fig. 3:**
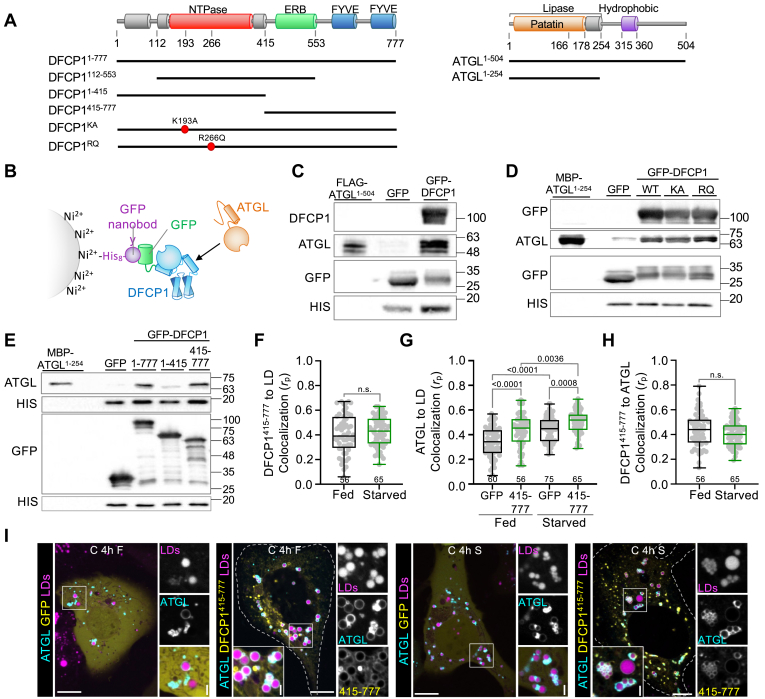
DFCP1 Interacts with ATGL. A: Domain diagrams of DFCP1 and ATGL. DFCP1 contains an N-terminal Zinc-finger-like domain, an NTPase domain, and ER-binding (ERB) domain that binds to both the ER and LDs, and a pair of C-terminal PI3P-binding FYVE domains (FYVE). ATGL contains an N-terminal patatin-like lipase domain and a long-disordered region containing an LD-association domain. Constructs used in these studies are shown below each domain diagram. B: Diagram of the semi-in vitro pulldown assay. GFP-DFCP1 is first pulled out of lysates using His-GFP nanobodies bound to Ni-NTA resin, and subsequently washed repeatedly with PBS. This resin is then used to examine the ability of DFCP1 to pull-down purified ATGL. C: Semi-in vitro pulldown of purified FLAG-ATGL using either GFP or GFP-DFCP1 constructs as bait, expressed in HEK 293T cells and pulled down onto HIS-tagged GFP-nanobody beads. D: Semi-in vitro pulldown of purified MBP-ATGL (1–254) using GFP or GFP-DFCP1^WT^, GFP-DFCP1^KA^, or GFP-DFCP1^RQ^ bound to HIS-tagged GFP-nanobody beads. E: Semi-in vitro pulldown of purified MBP-ATGL (1–254) using GFP, GFP-DFCP1^1-777^, GFP-DFCP1^1-415^, or GFP-DFCP1^415-777^ bound to HIS-tagged GFP-nanobody beads. F: Colocalization analysis (Pearson’s correlation coefficient) of GFP-DFCP1^415-777^ with LDs in fed (black) and starved (green) cells treated as in (I). G: Colocalization analysis of BFP-ATGL with LDs in fed or starved cells expressing GFP (black) or GFP-DFCP1^415-777^ (green) and treated as in (I). H: Colocalization analysis of GFP-DFCP1^415-777^ (green) with BFP-ATGL in fed (black) or starved (green) cells and treated as in I. I: Representative images of U2OS cells expressing either GFP or GFP-DFCP1^415-777^ and BFP-ATGL, treated with 200 μM OA for 20 h and then fed (basal growth media) or starved (EBSS) for 4 h, and treated with LipidTOX Deep Red for 30 min prior to imaging. The scale bars in whole-cell and inset images represent 10 and 2 μm, respectively. The statistical significance of the measurements in (F–H) was determined using the Mann–Whitney U-test on the indicated number of observations from three independent transfections. Exact *P*-values are reported with exception to *P* > 0.05, which is not considered to be significant (n.s.).

The localization experiments suggest that DFCP1 may directly interact with ATGL. To test this possibility, we set out to pull down purified human ATGL using GFP-nanobody conjugated beads bound to GFP-DFCP1 (Fig. 3A, B). Using this approach, we found that GFP-DFCP1 was not only able to pull down purified full-length ATGL (Fig. 3C and supplemental Fig. S3A), but also an N-terminal truncation of ATGL (residues 1–254) fused to maltose-binding protein (MBP-ATGL^1-254^) that contains the patatin-like lipase domain (Fig. 3D and supplemental Fig. S3B). Interestingly, this region of ATGL plays a central role in its interactions with other ATGL interactors, such as CGI-58 (30). Using this more tractable fragment of ATGL that can be purified to a higher degree, we determined that mutations in the NTPase domain of DFCP1 do not impair the interaction with ATGL since DFCP1^KA^ pulls down with ATGL to the same extent as DFCP1^WT^ (Fig. 3D), which is in strong agreement with our colocalization experiments (Fig. 2K, L). Additionally, we found that a second mutation (R266Q) in the NTPase domain of DFCP1 (Fig. 3A), which drives accumulation of DFCP1 on LDs (9), was also able to pull down ATGL to the same extent as DFCP1 (Fig. 3D). Since these NTPase domain mutations do not influence binding of DFCP1 to ATGL, we conclude that the NTPase domain is not necessary for the physical interaction between ATGL and DFCP1 but remains important for the localization of DFCP1 to LDs. Indeed, by performing a systematic truncation analysis (Fig. 3A), we found that the N-terminal 415 residues of DFCP1 that include the NTPase domain, DFCP1^1-415^, were not able to pull down ATGL (Fig. 3E). By contrast, truncations that contain the C-terminal FYVE domains, DFCP1^415-777^ or DFCP1^554-777^ were able to pull down ATGL (Fig. 3E). Thus, the interaction between ATGL and DFCP1 requires the N-terminal lipase domain of ATGL and the C-terminal FYVE domains of DFCP1.

To validate this interaction, we set out to determine if the colocalization of ATGL with LDs was influenced by the minimal LD-interacting fragment DFCP1^415-777^. This truncation localized well to LDs on its own but this localization was insensitive to starvation (Fig. 3F, I), which is likely due to the absence of the NTPase domain (9). The coexpression of GFP-DFCP1^415-777^ with BFP-ATGL led to an increase in LD localization of ATGL in both fed and starved cells when compared to the expression of GFP alone (Fig. 3G, I). Interestingly, despite GFP-DFCP1^415-777^ not showing a significant increase in LD localization upon starvation (Fig. 3F) or change in its colocalization with ATGL (Fig. 3H), there was a marked increase in the BFP-ATGL accumulation on LDs in starved cells when compared to fed cells (Fig. 3G). This suggests that this DFCP1^415-777^ may synergize with ATGL and/or other ATGL activating factors to promote the accumulation of ATGL on LDs.

### DFCP1 anchors ATGL to the LD

**Fig. 4:**
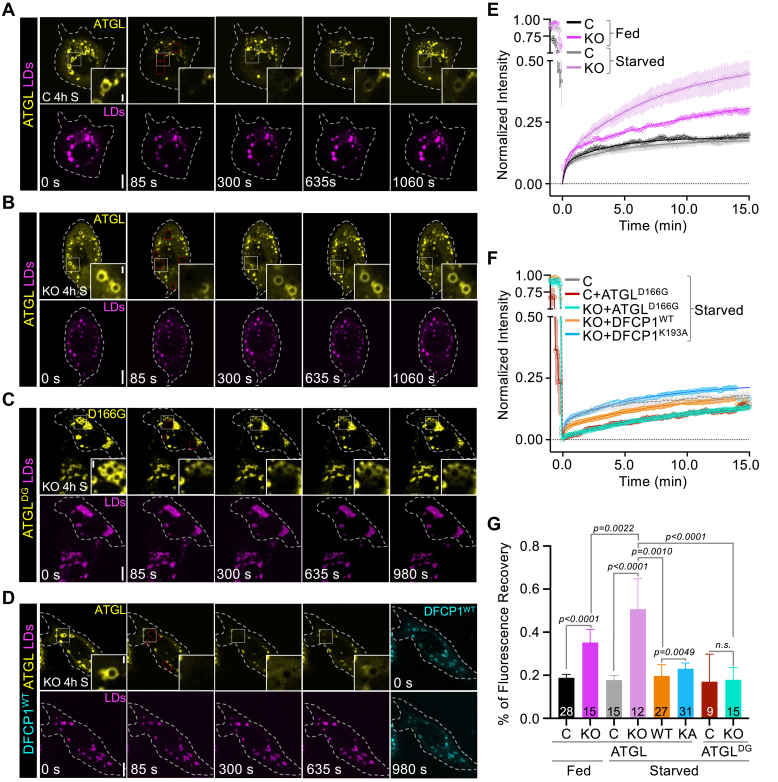
DFCP1 Anchors ATGL to the LD. A: Fluorescence Recovery after Photobleaching (FRAP) of GFP-ATGL on individual LDs (shown in the inset) in control U2OS cells that were stimulated with 200 μM OA for 20 h. Cells were then starved (EBSS) for 4 h and treated with LipidTOX Deep Red for 30 min prior to imaging. B: FRAP of GFP-ATGL on individual LDs (shown in the inset) in DFCP1 KO U2OS cells that were treated as in (A). C: FRAP of GFP-ATGL^DG^ on individual LDs (shown in the inset) in DFCP1 KO U2OS cells that were treated as in (A). D: FRAP of GFP-ATGL on individual LDs (shown in the inset) in DFCP1 KO U2OS cells rescued with BFP-DFCP1 and treated as in (A). E: Normalized average fluorescence recovery traces of GFP-ATGL in fed control cells (black), starved control cells (gray), fed DFCP1 KO cells (magenta), and starved DFCP1 KO cells (light magenta). Data is presented as mean ± SEM for the indicated number of traces (reported in G) and overlayed with a least-squares 2-component fit of the data (see methods). F: Normalized average fluorescence recovery traces of GFP-ATGL^DG^ in starved control (brick red) and DFCP1 KO cells (cyan) and GFP-ATGL in starved DFCP1 KO cells rescued with BFP-DFCP1^WT^ (orange) or BFP-DFCP1^KA^ (blue). Data is presented and fitted as in (E). G: Extent of fluorescence recovery of GFP-ATGL^WT^ and GFP-ATGL^DG^ on LDs 15 min after photobleaching in fed (black) and starved (gray) control cells, fed (magenta) and starved (light magenta) KO cells, starved KO cells rescued with BFP-DFCP1^WT^ (orange) or BFP-DFCP1^KA^ (blue), and starved control (brick red) or KO (cyan) cells expressing GFP-ATGL^DG^. All scale bars in full cell and inset images represent 10 and 2 μm, respectively. The statistical significance of the measurements in (G) was determined using a two-tailed parametric student *t* test based on the indicated number of observations. Exact *P*-values are reported with exception to *P* > 0.05, which is not considered to be significant (n.s.).

The dynamics of ATGL with LDs have been previously shown to correlate with the cellular activity of ATGL, such that activated ATGL associates and dissociates on LDs more quickly than inactive ATGL (31). Since DFCP1 influences the localization of ATGL in cells, we therefore wondered if DFCP1 could also impact ATGL dynamics, and, consequently, its activity. To address this question, we examined dynamic changes of GFP-ATGL on LDs using fluorescence recovery after photobleaching (FRAP), in fed and starved U2OS cells (Fig. 4 and supplemental Fig. S4). We found that GFP-ATGL in fed (supplemental Fig. S4A and supplemental Movie S1) and starved (Fig. 4A and supplemental Movie S2) control cells recovered with a similar biphasic rate and to a similar extent (19% vs. 18%) 15 min after photobleaching (Fig. 4A, E, G). By contrast, GFP-ATGL also recovered in a biphasic manner, but to a greater extent in both fed (35%) and starved (51%) DFCP1 KO cells, with the recovery of ATGL being significantly more in starved KO cells at 15 min after photobleaching (Fig. 4B, E, G, supplemental Fig. S4B, and supplemental Movies S1 and S2). This increase in GFP-ATGL dynamics likely reflects an increase in ATGL activity in the absence of DFCP1, since a kinase-dead mutant of GFP-ATGL^DG^ showed little fluorescence recovery to LDs in both starved control and DFCP1-KO cells (Fig. 4C, F, G, supplemental Fig. S4C, and supplemental Movie S3).

This enhancement of ATGL activity is specific to DFCP1 since rescuing DFCP1 KO cells with BFP-DFCP1 reduced the extent of recovery to that of control cells (Fig. 4D, F, G, and supplemental Movie S4). Rescuing DFCP1 KO cells with DFCP1^KA^ also reduced the extent of GFP-ATGL recovery, but not to the same extent as WT DFCP1 (Fig. 4F, G, supplemental Fig. S4D and supplemental Movie S4). It should be noted that in these rescue experiments, we only examined the recovery of GFP-ATGL on LDs that were also coated with DFCP1, and thus this analysis does not account for the impaired localization of DFCP1^KA^ with LDs (supplemental Fig. S2H, I).

As suggested by the fluorescence recovery at 15 min, the rate of association for ATGL to LDs is markedly faster in DFCP1 KO cells. The average recovery trace for all conditions with GFP-ATGL fits well to a two-component association model (see methods). The first association rate was rapid and has a similar *t*_*1/2*_ (∼0.25 min) across all conditions, which accounts for a small fraction (0.5%–30%) of the total recovery. This fast recovery phase likely represents the fluorescence recovery of a cytosolic fraction of GFP-ATGL (supplemental Table S1). By contrast, the second association rate was slow (supplemental Fig. S4E), and likely represents recovery of GFP-ATGL to the surface of LDs. However, the large variability in the *t*_*1/2*_ values suggests that the mobile fraction is not well defined at 15 min. This was in part due to the difficulty in tracking photobleached LDs beyond 15–20 min and thus we were unable to experimentally determine the true final fluorescence recovery value. Therefore, by assuming that GFP-ATGL can recover to the same extent as that seen in starved DFCP1 KO cells (51%), we found that the rate of GFP-ATGL was slow for control and DFCP1 rescue cells and was similar to the recovery of the enzymatically dead GFP-ATGL^DG^. By contrast, GFP-ATGL recovery was markedly faster for fed (*t*_*1/2*_ = 16.2 ± 2.0 min) and starved (*t*_*1/2*_ = 5.4 ± 0.38 min) DFCP1 KO cells (supplemental Fig. S4F). Additionally, DFCP1 KO cells rescued with BFP-DFCP1^KA^, showed a slightly more rapid recovery of GFP-ATGL than cells rescued with BFP-DFCP1 (*t*_*1/2*_ = 27.7 ± 4.8 min vs. 33.2 ± 6.8 min). Taken together, this analysis suggests that DFCP1 functions as a tether that slows down the dynamic exchange of ATGL on the surface of LDs.

### DFCP1 inhibits ATGL-dependent hydrolysis of TAGs

**Fig. 5:**
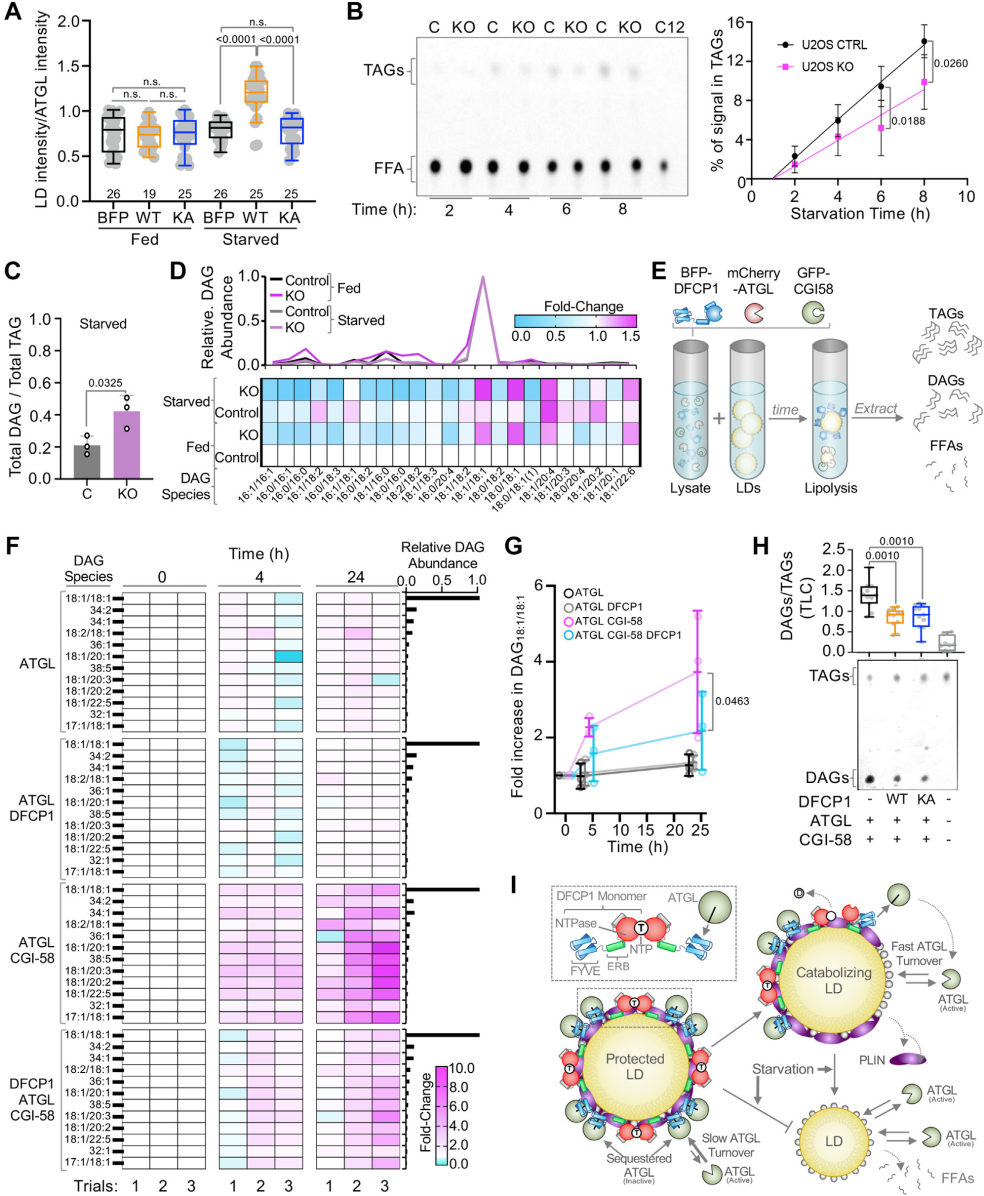
DFCP1 Inhibits ATGL-Dependent Hydrolysis of TAGs. A: LD content in U2OS cells expressing GFP-ATGL and either BFP, BFP-DFCP1^WT^ or BFP-DFCP1^KA^ and treated with 200 μM OA for 20 h. Cells were then fed or starved for 4 h and treated with LipidTOX Deep Red for 30 min prior to imaging. Sum projections of each Z-stack of entire cells were taken and total LD intensity for each cell was normalized to intensity of ATGL in the cell. B: TLC plate showing the conversion of Bodipy C12 labeled TAGs into free Bodipy C12 FAs in control and DFCP1 KO U2OS cells that were treated with 2 μM Bodipy C12 in EBSS for the indicated time interval. The C12 lane shows free Bodipy C12 blotted on the TLC plate immediately before mobilization. Quantification of the accumulation of TAG levels in control and DFCP1 KO cells for 3 independent experiments is shown on the right. C: Total DAGs normalized to the total TAGs found in LDs purified from U2OS control (C) and DFCP1 KO (KO) cell that were treated with 200 μM OA for 20 h and starved for 4 h. D: Heatmap showing the enrichment or loss of individual DAG species from LDs purified from U2OS control and DFCP1 KO cells in (C), relative to those DAGs found in LDs isolated from WT fed cells. The plot above the heatmap shows the average fraction of each DAG species relative to the most abundant DAG (DAG 18:1/18:1) found in the LDs for each condition. E: Diagram for reconstitution of LD lipolysis. LDs isolated from DFCP1 KO U2OS cells were incubated for 0–24 h with clarified lysates from HEK 293T cells expressing different combinations of GFP, GFP-CGI-58, mCherry, mCherry-ATGL, and either BFP, BFP-DFCP1^WT^, or BFP-DFCP1^KA^. Neutral lipids were extracted and analyzed using mass spectrometry (F) and thin layer chromatography (H). F: Heatmap showing the time-dependent evolution of DAGs in reactions depicted in (E). LDs isolated from DFCP1 KO cells mixed with lysates containing the following: BFP, GFP and mCherry-ATGL (top); BFP-DFCP1, GFP, mCherry-ATGL (second from top); BFP, GFP-CGI-58, and mCherry-ATGL (second from bottom); BFP-DFCP1, GFP-CGI-58, and mCherry-ATGL. The fold-change of the top 12 detected DAG species at 4 and 24 h, relative to their abundance at 0 h for each trial is shown in the heatmap. Each trial represents an independent preparation of LDs mixed with an independent preparation of lysates. The relative average abundance of each DAG at 24 h, relative to the total DAG pool at 24 h, normalized to the most abundant DAG (DAG 18:1/18:1) is shown in the horizontal bar graphs on the right of each experiment. G: The fold-change of DAG 18:1/18:1 for each trial of the 4 experiments in (F) as a function of time. Each open circle represents the fold-change of each trial relative to its 0 h value (normalized to 1). H: Reconstitution of LD lipolysis using TLC. LDs purified from DFCP1 KO U2OS cells were incubated with clarified HEK 293T lysates expressing GFP-CGI-58, mCherry-ATGL, and either BFP, BFP-DFCP1^WT^, or BFP-DFCP1^KA^ for 16 h and the resulting neutral lipid species were separated on TLC. Quantification of DAGs normalized to TAGs is shown on top. I: Model of the mechanism by which DFCP1 regulates ATGL. Upon starvation, DFCP1 promotes the recruitment of ATGL to the LD surface through an interaction between the N-terminal catalytic domain of ATGL with the DFCP1^415-777^. This interaction immobilizes ATGL on the LD surface and represses its activity. Loss of DFCP1 or an unknown release mechanism frees ATGL and allows it to rapidly dissociate and reassociate with LDs, to promote their catabolism. The statistical significance of the measurements in (A, C, and H) was determined using an unpaired parametric student *t* test on the indicated number of cells from three independent transfections for each condition in (A), three independent experiments for the two conditions in (C), three independent experiments (H) (all technical replicates from the 3 independent experiments are plotted). The statistical significance of the measurements in (B and G) were determined using a paired parametric student *t* test on three independent trials for each experimental condition. Exact *P*-values are reported with exception to *P* > 0.05, which is not considered to be significant (n.s.).

We have shown that DFCP1 drives the localization and dynamics of ATGL. Whereas this is likely part of the mechanism that DFCP1 uses to regulate LD size, we cannot exclude that DFCP1 can also be a negative regulator of ATGL activity. To address this question, we first investigated whether overexpression of DFCP1 could protect LDs from ATGL-mediated degradation in cells. For this purpose, we examined the LD content in fed and starved U2OS cells overexpressing GFP-ATGL, normalized to the ATGL intensity. We normalized to ATGL intensity because it is well appreciated that the amount of LDs is inversely correlated with the abundance of ATGL (32). We found that transient expression of either BFP-DFCP1^WT^ or BFP-DFCP^KA^ did not lead to a significant increase in LDs (based on the intensity of the LD marker LipidTOX) relative to GFP-ATGL expression levels (based on the intensity of GFP-fluorescence) (Fig. 5A). However, during starvation, BFP-DFCP1^WT^, but not BFP or BFP-DFCP1^KA^, promoted an accumulation of LDs (Fig. 5A). Thus, DFCP1^WT^ overexpression can help to protect LDs, even when ATGL is overexpressed.

To determine if DFCP1 impairs lipolysis by ATGL in cells, we used thin-layer chromatography (TLC) to compare the time-dependent increase of TAGs in starved control and DFCP1 KO cells stimulated to form LDs with a fluorescent lipid, Bodipy-C12 (Fig. 5B). When compared to starved control cells, starved DFCP1 KO cells showed a reduced time-dependent accumulation of TAGs, which is consistent with an increase in lipolysis to counteract the storage of TAGs. To further support this assertion, we examined the lipidomic profiles of LDs purified from OA-stimulated control and KO cells (Fig, 5C, D, and supplemental Fig. S5A, B). Under starved conditions, there was a significant increase in the total amount of all observed DAG species relative to the total amount of all observed TAG species in KO cells, when compared to control cells (Fig. 5C). There was also a similar pattern of enrichment and de-enrichment of specific TAGs and DAGs in both fed and starved KO cells, that was distinct from fed and starved control cells (Fig. 5D and supplemental Fig. S5A). In particular, the most abundant DAG (18:1/18:1) - formed from the hydrolysis of the most abundant TAG (18:1/18:1/18:1) resulting from OA stimulation - was acutely enriched in both fed and starved KO cells, with starved KO cells showing the most enrichment of this species. Furthermore, we did not observe any changes in the abundances of cholesterol esters across all conditions (supplemental Fig. S5B), which further supports that DFCP1 specifically regulates ATGL and not neutral lipid storage in general.

In cells, the activity of ATGL is enhanced by the LD resident protein, CGI-58, and thus we wanted to more directly test if DFCP1 can regulate a reconstituted physiologically relevant lipolytic reaction. To do so, we developed a semi-in vitro assay to assess how the lipid landscapes of LDs would change when mixed with a combination of ATGL, CGI-58 and DFCP1 (Fig. 5E). To that end we incubated LDs isolated from OA-stimulated DFCP1-KO cells with clarified HEK293T lysates of cells transiently expressing the following combinations: (1) BFP, GFP and mCherry-ATGL; (2) BFP-DFCP1^WT^, GFP, and mCherry-ATGL; (3) BFP, GFP-CGI58, and mCherry-ATGL, or (4) BFP-DFCP1^WT^, GFP-CGI58, and mCherry-ATGL. Importantly, the critical proteins (DFCP1, ATGL and CGI-58) in these reactions expressed to a similar extent across the 5 lysates (supplemental Fig. S5C). Using mass-spectrometry on the lipids extracted from these reactions, we found that ATGL or the combination of ATGL and DFCP1 had little impact on the rate of formation of the 12 most abundant DAGs (Fig. 5F) or the hydrolysis of the 15 most abundant TAGs (supplemental Fig. S5D). By contrast, the inclusion of CGI-58 with ATGL had a massive impact on the rate of DAG formation, which was impaired when DFCP1 was included with CGI-58 and ATGL (Fig. 5F). In particular, the most abundant DAG (DAG 18:1/18:1) increased ∼2- to 5-fold (depending on the trial) when DFCP1 was absent, but either slightly decreased or increased up to ∼2 fold when DFCP1 was present (Fig. 5G). By comparison, changes in the rate of TAGs were considerably less dramatic and more variable (supplemental Fig. S5D). Consequently, we did not observe a significant change in the most abundant TAG (TAG 18:1/18:1/18:1) over 24 h in these reactions (supplemental Fig. S5E). This is likely due to the super abundances of TAGs found in the OA-stimulated LDs at the start of these reactions, which makes it difficult to deduce the relatively small changes that occur to this pool of TAGs over 24 h using mass-spectrometry. By contrast, LDs isolated from fed DFCP1 KO cells show relatively few DAGs initially (supplemental Fig. S5F).

To better assess the extent of TAG hydrolysis, we performed the same assay but assessed lipid content using TLC on chloroform-extracted lipids (Fig. 5H, and supplemental Fig. S5F, G). In this case, we compared the impact of BFP-DFCP1^WT^ and the DFCP1 hydrolysis mutant (BFP-DFCP1^KA^) on lipolysis by ATGL and CGI-58. The addition of lysates from cells overexpressing ATGL and CGI-58 showed a marked increase in 2,3 DAG, but not 1,3 DAG (supplemental Fig. S5F). Consistent with our previous observations, cell lysates containing similar abundances of DFCP1^WT^ or DFCP1^KA^ (supplemental Fig. S5G) had significantly lower levels of these DAGs, when normalized to the abundance of TAGs, indicating that DFCP1 directly impairs lipolysis by CGI-58-activated ATGL (Fig. 5H). However, unlike the cellular context (Fig. 5A), DFCP1^KA^, impaired lipolysis to the same extent as DFCP1^WT^, which is consistent with our pulldowns that show the interaction between DFCP1 and ATGL does not depend on the nucleotide state (Fig. 3C). In other words, the inability for DFCP1^KA^ to stimulate LD accumulation in cells is likely due to its mislocalization to other cellular compartments that are likely absent in the clarified lysates used in the assay. Altogether, this data presents strong evidence that DFCP1 is both a critical recruitment factor and repressor of ATGL-dependent hydrolysis of TAGs.

## Discussion

LDs serve as vital energy reservoirs that can be readily tapped to meet the energy demands of the cell. Therefore, it is imperative that the TAGs housed within LDs can be rapidly broken down into FFAs to fuel ATP synthesis by mitochondria. ATGL is the rate-limiting enzyme involved in the first step of TAG hydrolysis, however, little is known about the molecular factors that regulate this enzyme. In this study, we expanded upon the emerging functions of DFCP1 on LDs to show that it is a regulator of ATGL-mediated lipolysis (Fig. 5I). We show that loss of DFCP1 does not impact the LD biogenesis pathway, but mainly impairs pathways associated with LD catabolism (Fig. 1 and supplemental Fig. S1). In particular, DFCP1 controls the recruitment of ATGL to LDs (Fig. 2 and supplemental Fig. S2), which involves binding between the N-terminal patatin-like domain of ATGL and the C-terminal FYVE domains of DFCP1 (Fig. 3 and supplemental Fig. S3). This interaction anchors ATGL to the LD surface, thereby preventing dynamic turnover of ATGL necessary for its lipolytic function (Fig. 4 and supplemental Fig. S4). In line with this observation, loss of DFCP1 markedly enhances ATGL mediated breakdown of LDs (Fig. 5 and supplemental Fig. S5). When taken together, our data suggest that DFCP1 serves to prime ATGL for rapid LD catabolism by a two-step mechanism that consists of specific recruitment of ATGL to LDs by DFCP1, followed by inhibition of ATGL’s activity by DFCP1 (Fig. 5I).

The discovery that DFCP1 regulates LD metabolism by regulating the recruitment and function of ATGL is a departure from previous observations that linked DFCP1 to the biogenesis of LDs (10, 16). In that work, it was demonstrated that DFCP1 acts at the initial stages of LD biogenesis (10, 16), possibly through Seipin (16). However, our results suggest that DFCP1 is instead playing a role in LD catabolism by regulating both the recruitment and activity of ATGL. While these two roles of DFCP1 are not mutually exclusive, it has been shown that ATGL deficiency results in a compensatory loss of enzymes involved in de novo lipogenesis, including acetyl-CoA carboxylase (ACC1), fatty acid synthase (FAS), ATP-citrate lyase (ATP-CL), and acetyl-CoA synthetase (AceCS1) (33), which ultimately lead to impaired LD biogenesis. Additionally, depending on how ATGL is activated, DFCP1 could impact LD biogenesis under a specific context, such as during basal lipolysis. ATGL produces mostly *sn*-1,3 DAGs, but CGI-58 alters ATGL’s stereoselectivity to produce *sn*-1,3 and *sn*-2,3 DAGs. Since DGAT2 has a preference for sn-1,3 DAGs, this suggests that DGAT2 may re-esterify DAGs during basal lipolysis to reduce cytotoxicity, whereas DGAT1 may be important for both basal and CGI-58-stimulated lipolysis (34). Thus, DFCP1 may play more of a selective role in LD biogenesis during basal lipolytic breakdown. In any case, regulation of ATGL by DFCP1 is central to both the changes in LD biogenesis and catabolism.

Our observation that DFCP1 regulates LD dynamics through sequestration and inhibition of ATGL further raises the question how this DFCP1-ATGL interaction is released. One possibility is that it may compete with CGI-58 for interaction with ATGL. CGI-58 is arguably the best-studied regulatory protein of ATGL where it has been established to play a key role in enhancing both the rate and stereoselectivity of TAG hydrolysis (34). CGI-58 is known to be sequestered on the surface of LDs under basal conditions by perilipins (35, 36). Upon lipolytic stimulation, this repression is relieved through a phosphorylation-dependent mechanism (26) and CGI-58 can bind to LD-bound ATGL (36, 37). Consequently, this interaction greatly accelerates the in vivo activity of ATGL (8). Thus, it is possible that in addition to regulating the recruitment of ATGL, DFCP1 may prevent or disrupt the CGI-58-ATGL interaction on LDs. Indeed, DFCP1 can still inhibit ATGL activity even in the presence of CGI-58 in vitro (Fig. 5F, H). In this context, DFCP1 may mirror the function of perilipin 5 (Plin5/PLPN5) in cardiomyocytes, where Plin5 was shown to recruit ATGL to LDs (38) and sequester it from interacting with CGI-58 (37). Interestingly, Plin5 can interact with both CGI-58 and ATGL, but not both at the same time. Rather, Plin5, which oligomerizes, can partition both CGI-58 and ATGL on LDs and thereby prime lipolysis by concentrating these proteins on the LD surface (27). Similarly, DFCP1 can also oligomerize and could help to partition both ATGL and CGI-58 on the LD surface, to ready them for rapid LD catalysis. Since Plin5 is mainly expressed in highly oxidative tissues, it is possible that DFCP1 is a functional replacement in less oxidative tissues.

Another possibility is that DFCP1 may release ATGL in a nucleotide-dependent manner. We have previously shown that mutations in the NTPase domain of DFCP1 modifies the localization of DFCP1 to both LDs and autophagosomes (9). Indeed, a mutation that prevents the binding of nucleotide (DFCP1^KA^), leads to a loss of DFCP1 on LDs, whereas a mutation that promote dimerization (DFCP1^RQ^) leads to the stabilization of DFCP1 on the LD surface. Despite this, both mutant forms of DFCP1 can pull-down ATGL equally well. Additionally, if DFCP1^KA^ is on LDs, it can repress the dynamics of ATGL in cells (Fig. 4F, G, supplemental Fig. S5D and supplemental Movie S4) and the activity of ATGL in vitro (Fig. 5H). However, in cells, DFCP1^KA^ does not accumulate as well as DFCP1^WT^ on LDs (supplemental Fig. S2H) and therefore DFCP1^KA^ is less able to drive ATGL accumulation on LDs (Fig. 2I). Thus, regulation of ATGL by DFCP1 depends mainly on whether DFCP1 is on LDs, and NTPase mutations can directly influence lipolysis by ATGL by modifying the localization of DFCP1. At this time, it is unclear what factors stimulate DFCP1 catalytic activity, but autophagy-induced changes may play a role (9).

ATGL, like DFCP1, also plays a role in autophagy. Specifically, ATGL was shown to increase autophagy by promoting SIRT1 activity (39, 40). Furthermore, ATGL increases the accumulation of LC3 on LDs and enhances autophagic flux in the liver, which implies that ATGL impacts lipophagy as well as lipolysis (41). ATGL has also been suggested to be an autophagy adaptor, due to its potential LC3-interacting region (LIR) motifs where mutation of this motif displaces ATGL from the LD and disrupts lipolysis (42). As DFCP1 KD exhibits a modest effect on lipophagy (Fig. 1F) and has been shown to localize to sites of autophagosome biogenesis- even in the presence of LDs (9), it is possible that DFCP1 inhibits ATGL specifically at certain populations of LDs tethered to the ER to coordinate lipophagy and lipolysis. Further study will be required to fully investigate this possibility.

In summary, we have shown that DFCP1 is a negative regulator of ATGL-mediated lipolysis. Specifically, we show that DFCP1 recruits and subsequently sequesters ATGL on LDs. However, under conditions that promote translocation of DFCP1 away from LDs, ATGL is able to drive rapid lipolysis of LD (Fig. 5I). Thus, we speculate that DFCP1 functions as a nutrient-sensitive molecular switch that can promote LD growth under basal conditions but potentiates LD catabolism during starvation.

## Data availability

Data supporting LD analysis, FRAP analysis, and LD lipidomics are publicly available at the Kast Lab Dataverse (https://doi.org/10.7910/DVN/IJOUDG). Raw cell images are available upon request.

## Supplemental data

This article contains supplemental data.

## Conflict of interests

The authors declare that they have no conflicts of interest with the contents of this article.

## Supplementary data

Supplemental Information Legends

Figures JLR REVISION SUPP FIGS
