# Supplementary material for: DFCP1 is a regulator of starvation-driven ATGL-mediated lipid droplet lipolysis
Source: J Lipid Res. 2024 Nov 19;66(1):100700. doi: 10.1016/j.jlr.2024.100700 (PMC11721518; doi:10.1016/j.jlr.2024.100700)
Supplement: Supplemental Information Legends [file mmc1.docx]

**DFCP1 is a Regulator of Starvation-driven ATGL-mediated Lipid Droplet Lipolysis**

Victoria A. Ismail^1*^, Meg Schuetz^1*^, Zak N. Baker^1^, Jean A. Castillo-Badillo^1^, Teri V. Naismith^1^, David J. Pagliarini^1,2,3,4^ and David J. Kast^1†^

^1^Department of Cell Biology and Physiology, Washington University School of Medicine, St. Louis, Missouri 63110, USA.

^2^Howard Hughes Medical Institute, Chevy Chase, MD 20815, USA.

^3^Department of Biochemistry and Molecular Biophysics, Washington University School of Medicine, St. Louis, Missouri 63110, USA.

^4^Department of Genetics, Washington University School of Medicine, St. Louis, Missouri 63110, USA.

*Co-first authors

^†^Corresponding Author. Correspondence should be addressed to David J. Kast (kast@wustl.edu)

**Supplemental Table and Figure Legends**

**Supplemental Table 1: FRAP Fit Result.**

**Supplemental Figure 1: Interrogating the Role of DFCP1 in Lipid Metabolism**

**(A,B)** Number of LD density (**A**) and LD diameters (**B**) quantified from images of control and DFCP1 KD Hep3B cells expressing GFP that were treated with 200 μM OA for 20 h and starved (EBSS) for 18 h along with DMSO (vehicle) or the indicated inhibitors.

**(C)** Western blot showing the expression of DFCP1 in control and DFCP1 KO U2OS cells.

**(D)** Cell viability measurements taken from U2OS cells treated with 200 µM oleic acid (OA, left) or palmitic acid (PA, right) for 20 h and then fed (basal growth media) or starved (EBSS) for the indicated amount of time.

**(E)** Propidium iodide (left) or Annexin V (right) fluorescence normalized to DAPI fluorescence on U2OS cells treated with indicated amount of OA or PA for 20 h and then fed or starved for 24 h.

The statistical significance of the measurements in **A** and **B** was determined using an unpaired nonparametric student *t*-test (Mann–Whitney U-test) on the indicated number of observations from two independent transfections. Data from **D** and **E** are from three technical replicates from a single experiment. Comparisons between control and DFCP1 KO cells in all conditions are not statistically significant as determined using a two-tailed student *t*-test on the indicated number of observations. Exact *p*-values are reported with exception to *p*>0.05, which is not considered to be significant (n.s.).

**Supplemental Figure 2: Role of DFCP1 on Determinants of ATGL Localization.**

**(A)** Representative blots showing the typical purity of LDs used in **Figure 2C**. Importantly, the LD fraction has markedly reduced abundances of cytosolic proteins, such as GAPDH, and ER-resident proteins, such as Calreticulin.

**(B,C)** Representative blots of C2C12 (**B**) and 3T3L1 (**C**) mouse preadipocyte lysates extracted from control (black) and DFCP1 KO (magenta) cells treated with 200 μM OA for 20 h and then fed (basal growth media) or starved (EBSS) for 4 h. Densitometry of phosphorylated ATGL relative to 14-3-3 band is shown in the graph above the blots in **C**.

**(D)** Representative western blots showing PLIN3 (top) and PLIN5 (bottom) expression in fed and starved control (C, black) and DFCP1 KO (KO, magenta) clarified U2OS cell lysates. Relative PLIN3 abundances were determined by measuring the PLIN3 abundances using densitometry and normalizing to the measured 14-3-3 abundance. PLIN5 expression in U2OS cells was not clearly detectable using western blotting and therefore was not quantified.

**(E)** Representative images of DFCP1 KO U2OS cells expressing GFP-ATGL, rescued with either BFP (left), BFP-DFCP1^WT^ (middle), and BFP-DFCP1^KA^ (right). All cells were treated with 200 μM OA for 20 h before they were fed (basal growth media) and incubated with LipidTOX Deep Red for 30 min.

**(F)** Colocalization (Pearson’s correlation coefficient, *r_p_*) of GFP-ATGL with LDs, in fed KO cells rescued with either BFP (black), BFP-DFCP1^WT^ (WT, orange) and BFP-DFCP1^KA^ (KA, blue).

**(G)** Colocalization analysis (Pearson’s correlation coefficient, *r_p_*) of BFP-DFCP1^WT^ (WT, orange) and BFP-DFCP1^KA^ (KA, blue) with LDs in U2OS cells expressing GFP-ATGL and treated with 200 μM OA for 20 h before they were fed (basal growth media) or starved (EBSS) for 4 h.

**(H)** Representative images of U2OS cells expressing either GFP-DFCP1^WT^ or GFP-DFCP1^KA^. Cells were treated with 200 μM OA for 20 h, starved (EBSS) for 4 h, and incubated with LipidTOX Deep Red for 30 min.

**(I)** Colocalization analysis (Pearson’s correlation coefficient, *r_p_*) of GFP-DFCP1^WT^ (WT, black) and GFP-DFCP1^KA^ (KA, blue) with LDs in cells described in **H**.

The scale bars in whole-cell and inset images of **E** and **H** represent 10 and 2 µm, respectively. The statistical significance of the measurements in **C and D** was determined using a paired parametric student *t*-test on 5 independent experiments. The statistically significance of the measurements in **D** was determined using a paired parametric student *t*-test on 3 independent experiments, each consisting of an average of 3 technical replicates (all technical replicates are plotted in **D**). The statistical significance of the measurements in **F, G and I** was determined using an unpaired parametric student *t*-test on the indicated number of observations from two independent transfections. Exact *p*-values are reported with exception to *p*>0.05, which is not considered to be significant (n.s.).

**Supplemental Figure 3: Purity of Expressed ATGL Constructs.**

(**A, B**) Representative Coomassie-stained SDS-PAGE gels showing the purity of human full-length ATGL (**A**) and MBP-ATGL^1-254^ (**B**) expressed in and purified from HEK293T cells and E. coli, respectively.

**Supplemental Figure 4: Dynamics of ATGL and ATGL mutants on LDs.**

(**A, B**) Fluorescence Recovery after Photobleaching (FRAP) of GFP-ATGL on individual LDs (shown in the inset) in control and DFCP1 KO U2OS cells that were stimulated with 200 μM OA for 20 h and fed (basal growth media) for 4 h, with a subsequent treatment of LipidTOX Deep Red for 30 min.

**(C)** FRAP of kinase-dead mutant of ATGL (GFP-ATGL^DG^) on a single LD (shown in the inset) in control U2OS cells that were stimulated with 200 μM OA for 20 h and starved (EBSS) for 4 h.

(**D**) FRAP of GFP-ATGL on a single LD (shown in the inset) in DFCP1 KO cells that were rescued with BFP-DFCP1^KA^ and treated as in **C**.

(**E**) Time to half recovery (*t_1/2_*) for the slow rate of fluorescence recovery for GFP-ATGL^WT^ and GFP-ATGL^DG^ on LDs in fed (black) and starved (gray) control cells, fed (magenta) and starved (light magenta) KO cells, KO cells rescued with BFP-DFCP1^WT^ (WT, orange) or BFP-DFCP1^KA^ (KA, blue), and control (brick red) or KO (cyan) cells expressing GFP-ATGL^DG^. The slow rate was determined from least-squares 2-component “free” fit (see methods) of the data shown in Figures **4E** and **4F**. Data is presented as mean ±SD.

(**F**) Time to half recovery (*t_1/2_*) for the slow rate of fluorescence recovery from least-squares 2-component fits (see methods) of the averaged data in Figures **4E** and **4F**, where the mobile fraction for all traces was assumed to be the same as that found in starved DFCP1 KO cells. Columns are labeled as indicated in **E** and the data is presented as mean ±SD for the indicated number of traces.

All scale bars in whole cell and inset images represent 10 and 2 µm, respectively. The statistical significance of the measurements in **E** and **F** was determined using the Mann–Whitney U-test. Exact *p*-values are reported with exception to *p*>0.05, which is not considered to be significant (n.s.).

**Supplemental Figure 5: DFCP1 Inhibits ATGL-Dependent Hydrolysis of TAGs.**

**(A)** Heatmap showing the fold-increase or fold-decrease of all TAG species identified in LDs purified from fed and starved control and DFCP1 KO U2OS cells, relative to those TAGs found in LDs isolated from fed control U2OS cells. The plot above the heatmap shows the average abundance of each TAG species, relative to the most abundant TAG (18:1/18:1/18:1) species, found in the LDs harvested from condition.

(**B**) Heatmap showing the fold-increase or fold-decrease of all CE species from LDs purified described in A and relative to those CEs found in LDs isolated from fed control U2OS cells. The plot above the heatmap shows the average abundance of each CE species, relative to the most abundant CE (16:1/18:2) species.

**(C)** Representative HEK 293T lysates showing the expression levels of GFP-CGI-58 (detected using a GFP antibody), mCherry-ATGL (detected using an ATGL antibody), and BFP-DFCP1^WT^ (detected using a DFCP1 antibody) used in the mass spectrometry lipolytic rate assays (**Figure 5F and Supplemental Figure 5D)**.

(**D**) Heatmap showing the time-dependent evolution of TAGs in reactions consisting of isolated LDs from DFCP1 KO cells mixed with lysates containing the following: BFP, GFP and mCherry-ATGL (top); BFP-DFCP1, GFP, mCherry-ATGL (2^nd^ from top); BFP, GFP-CGI-58, and mCherry-ATGL (2^nd^ from bottom); and BFP-DFCP1, GFP-CGI-58, and mCherry-ATGL. The fold-change of the top 15 detected TAG species at 4 and 24 h, relative to their abundance at 0 h for each trial is shown in the heatmap. Each trial represents an independent preparation of LDs mixed with an independent preparation of lysates. The average abundance of each TAG at 24 h relative to the total TAG pool at 24 h and normalized to the most abundant TAG, is shown in the horizontal bar graphs on the right of each experiment.

(**E**). The fold-change of TAG 18:1/18:1/18:1 for each trial of the 4 experiments indicated in **D** as a function of time. Each open circle represents the fold-change of each trial relative to its 0 h value, which was normalized to 1.

**(F)** TLC plate showing lipase activity after 16 h of incubation of LDs from DFCP1 KO U2OS cells, mixed with HEK 293T lysates expressing GFP-CGI-58, mCherry-ATGL, and either BFP-DFCP1^WT^ (WT) or BFP-DFCP1^KA^ (KA). LD lane shows the amount of DAGs in LDs incubated with no HEK lysates. DAG lane is a control for the mobility of 1,3 DAGs and 2,3 DAGs.

**(G)** HEK 293T lysates showing the expression levels of GFP-CGI-58 (anti-GFP), mCherry-ATGL (anti-ATGL), and BFP-DFCP1^WT^ or BFP-DFCP1^KA^ (anti-DFCP1).

The statistical significance of the measurements in **E** was determined using a paired parametric student t-test on the three independent trials for each experimental condition shown in **D**. Exact *p*-values are reported with exception to *p*>0.05, which is not considered to be significant (n.s.).

**Supplemental Movie 1.** Representative FRAP videos of GFP-ATGL on individual LDs in fed control (left) and DFCP1 KO (right) U2OS cells that were stimulated with 200 μM OA for 20 h and incubated with LipidTOX Deep Red for 30 min prior to the experiment. Photobleached regions are indicated by the white boxes.

**Supplemental Movie 2.** Representative FRAP videos of GFP-ATGL on individual LDs in control (left) and KO (right) U2OS cells that were stimulated with 200 μM OA for 20 h and starved (EBSS) for 4 h and incubated with LipidTOX Deep Red for 30 min prior to the experiment. Photobleached LDs are indicated by the white boxes.

**Supplemental Movie 3.** Representative FRAP videos of kinase-dead mutant of ATGL (GFP-ATGL^DG^) in control (left) and KO (right) U2OS cells that were stimulated with 200 μM OA for 20 h, starved (EBSS) for 4 h, and incubated with LipidTOX Deep Red for 30 min prior to the experiment. Photobleached LDs are indicated by the white boxes.

**Supplemental Movie 4.** Representative FRAP videos of GFP-ATGL in DFCP1 KO U2OS cells rescued with either BFP-DFCP1^WT^ (left) or BFP-DFCP1^KA^ (right). Cells were stimulated with 200 μM OA for 20 h, starved (EBSS) for 4 h and incubated with LipidTOX Deep Red for 30 min prior to the experiment. Photobleached LDs are indicated by the white boxes.
