# Supplementary material for: DFCP1 is a regulator of starvation-driven ATGL-mediated lipid droplet lipolysis
Source: J Lipid Res. 2024 Nov 19;66(1):100700. doi: 10.1016/j.jlr.2024.100700 (PMC11721518; doi:10.1016/j.jlr.2024.100700)

Supplemental Figure 1

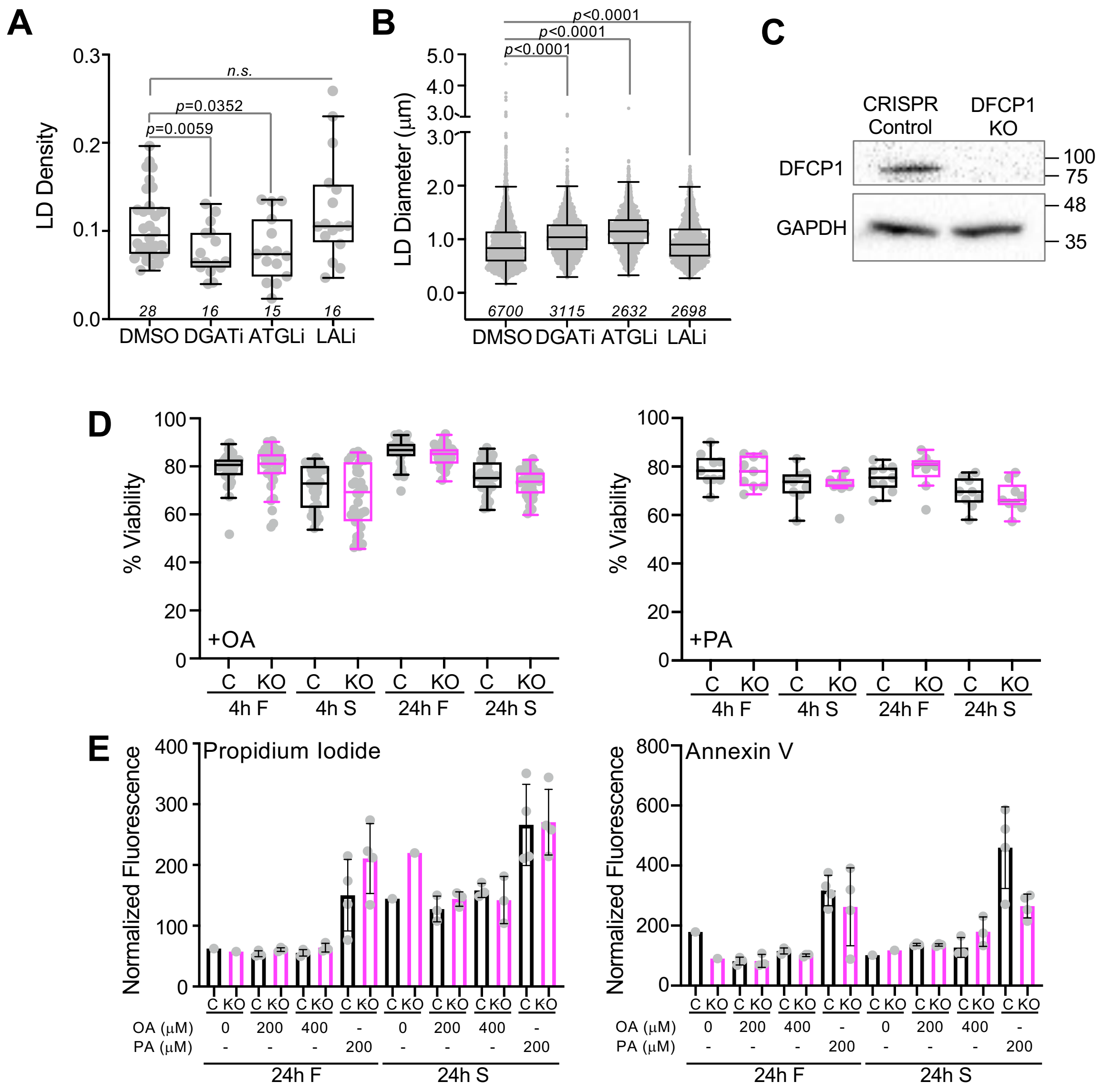

Supplemental Figure 2

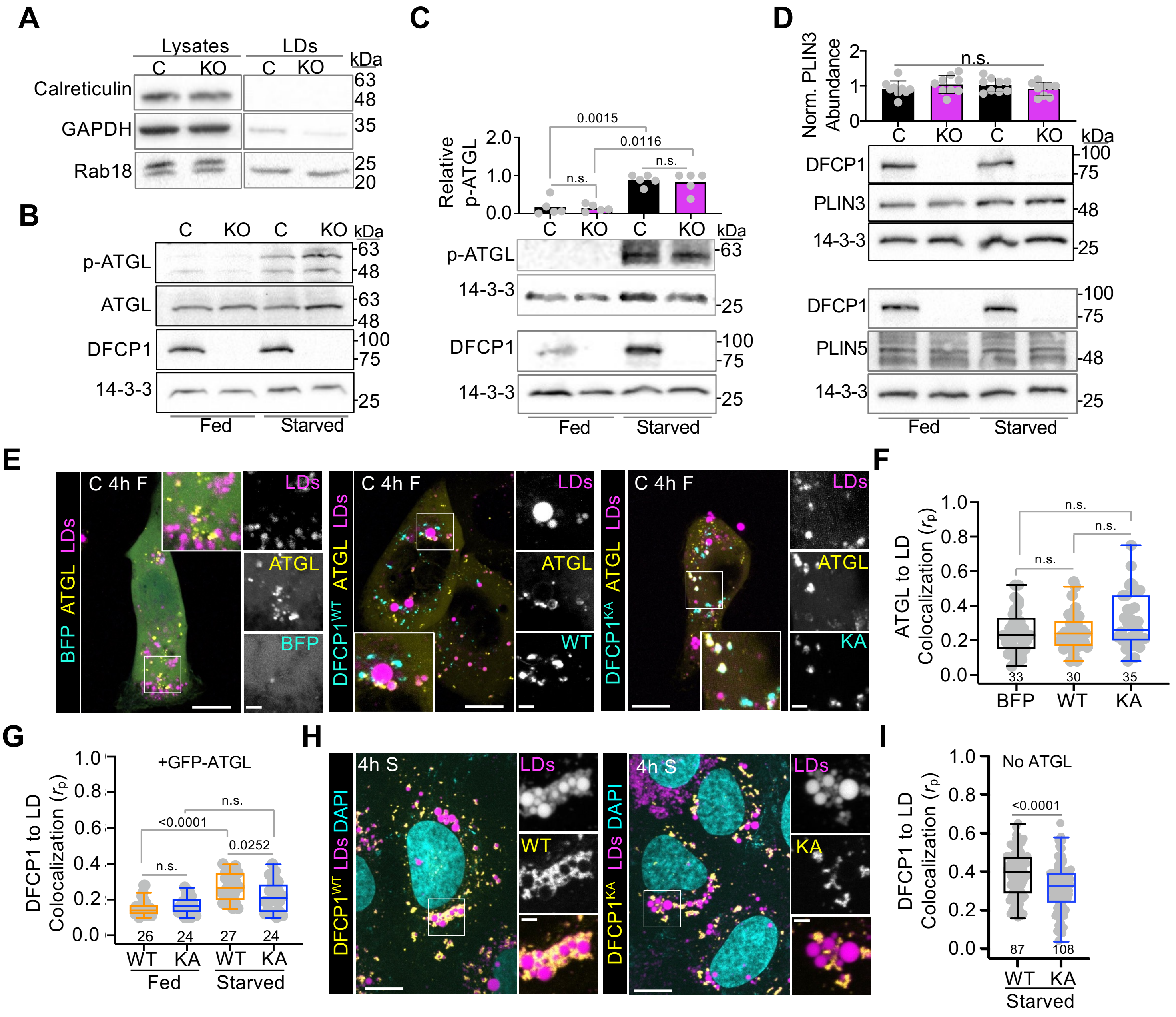

Supplemental Figure 3

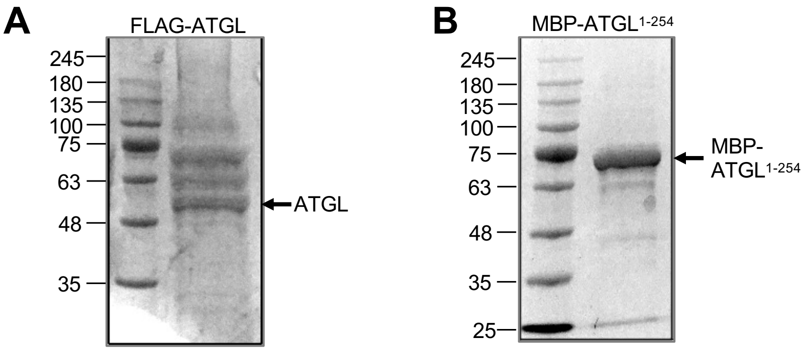

Supplemental Figure 4

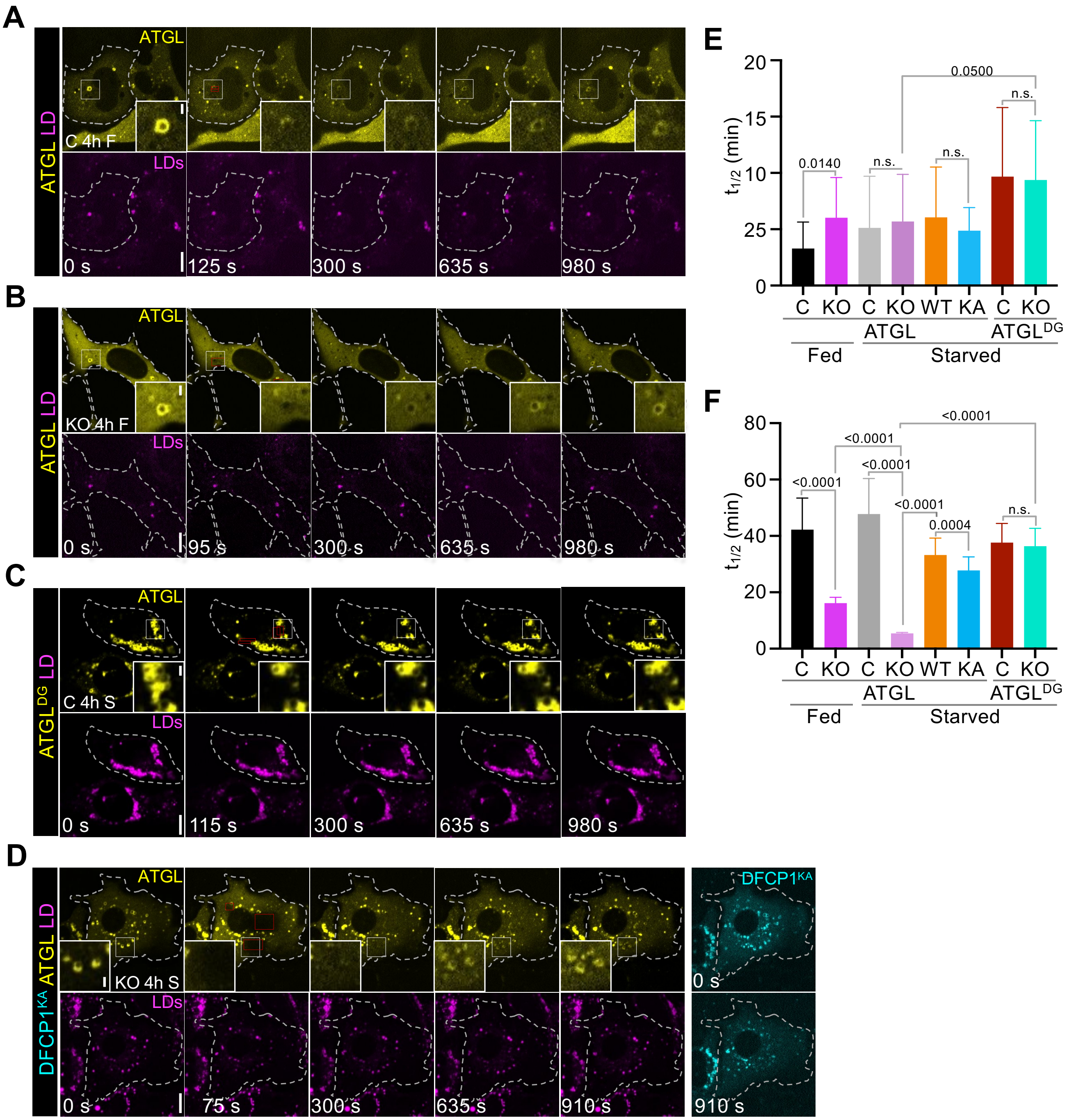

Supplemental Figure 5

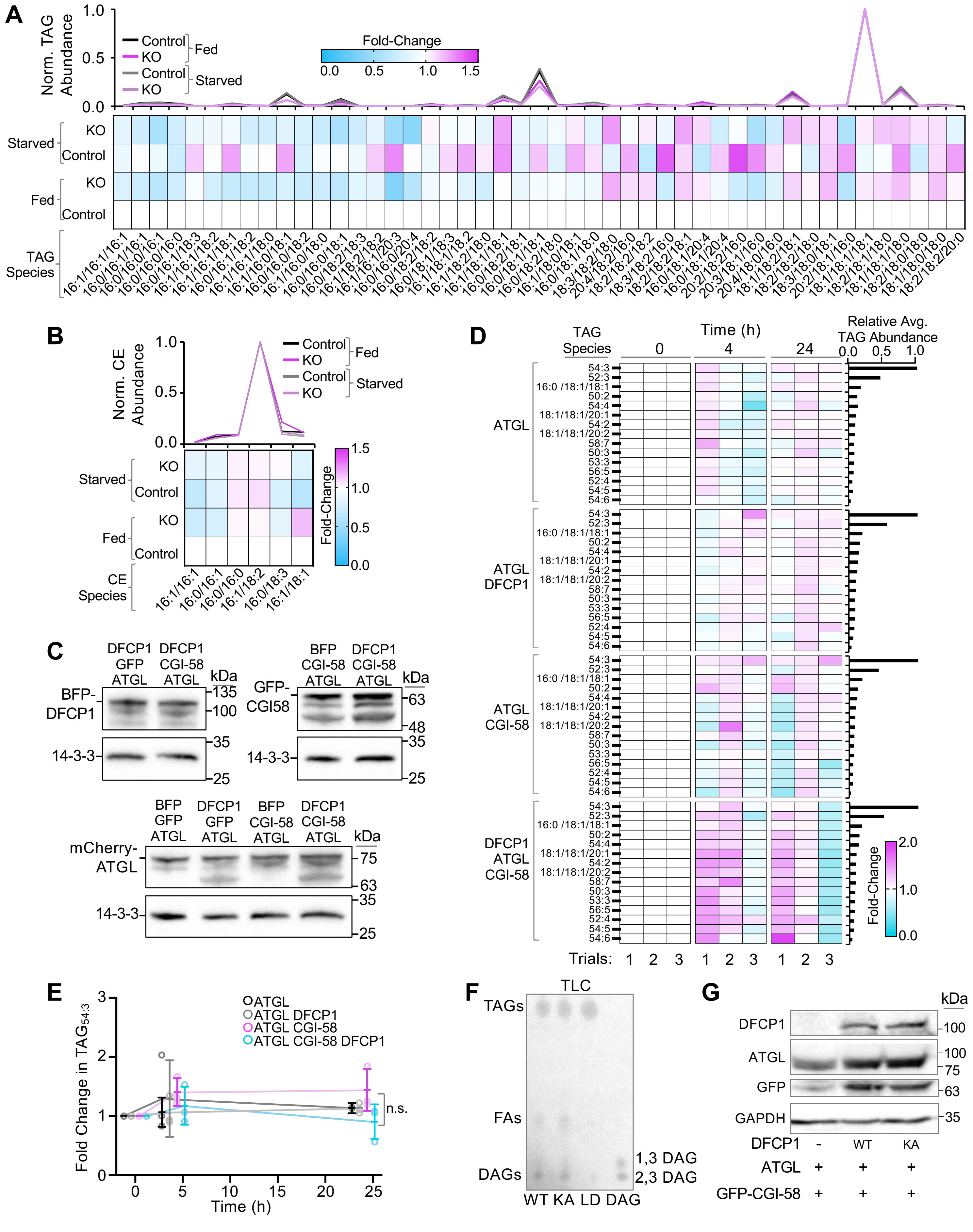

Supplement: Figures JLR REVISION SUPP FIGS [file mmc2.pdf]
