# Supplementary material for: DFCP1 is a regulator of starvation-driven ATGL-mediated lipid droplet lipolysis
Source: J Lipid Res. 2024 Nov 19;66(1):100700. doi: 10.1016/j.jlr.2024.100700 (PMC11721518; doi:10.1016/j.jlr.2024.100700)
Supplement: Supplemental Table 1 [file mmc3.pdf]

Table S1: FRAP Fit Results

Fit 1: 2-Component Association

	% Recovery		χ		k_f		k_s		RMSE
	Mean	SE	Mean	SE	Mean	SE	Mean	SE	
Fed Control	0.197	0.003	0.357	0.032	3.460	0.760	0.211	0.028	0.069
Starve Control	0.174	0.008	0.328	0.027	3.728	1.073	0.135	0.029	0.060
Fed KO	0.361	0.015	0.325	0.016	4.260	1.136	0.115	0.018	0.077
Starve KO	0.508	0.032	0.218	0.037	1.99	0.960	0.121	0.022	0.137
Starve KO+DFCP1	0.197	0.010	0.246	0.013	6.565	2.427	0.114	0.016	0.054
Starve KO+DFCP1^{K1993A}	0.230	0.005	0.332	0.009	6.962	1.220	0.142	0.011	0.041
Starve Control+ATGL^{D166G}	0.191	0.021	0.065	0.010	3.15	0.671	0.072	0.014	0.037
Starve KO+ATGL^{D166G}	0.187	0.015	0.080	0.010	6.59	6.021	0.074	0.011	0.035

Fit: 2-Component Association with Mobile set to 0.508

	% Recovery	χ		k_f		k_s		RMSE
	Mean	Mean	SE	Mean	SE	Mean	SE	
Fed Control	0.508	0.236	0.005	2.460	0.149	0.018	0.001	0.064
Starve Control	0.508	0.208	0.006	2.460	0.149	0.017	0.001	0.064
Fed KO	0.508	0.203	0.007	2.460	0.149	0.045	0.001	0.064
Starve KO	0.508	0.208	0.010	2.460	0.149	0.125	0.003	0.064
Starve KO+DFCP1	0.508	0.129	0.005	2.460	0.149	0.022	0.001	0.064
Starve KO+DFCP1^{K1993A}	0.508	0.200	0.005	2.460	0.149	0.026	0.001	0.064
Starve Control+ATGL^{D166G}	0.508	0.049	0.007	2.460	0.149	0.018	0.001	0.064
Starve KO+ATGL^{D166G}	0.508	0.044	0.006	2.460	0.149	0.019	0.001	0.064

2-Component Association Fit Model: $F(t) = F_R[\chi\left(1 - e^{-k_f t}\right) + (1-\chi)(1 - e^{-ik_s t})]$

χ: mol fraction

k_f: fast recovery rate

k_s: slow recovery rate

RMSE: Root Mean Square Error representing the goodness of fit
